# Dietary Supplement Use from Infancy to Adolescence in Croatia: Prevalence, Types and Associated Factors

**DOI:** 10.3390/children13101434

**Published:** 2026-10-19

**Authors:** Ivana Rumbak, Ana Ilić, Andrea Gradiška, Martina Pavlić, Lidija Šoher, Daniela Čačić Kenjerić, Jasna Pucarin-Cvetković, Darja Sokolić

**Affiliations:** 1 Department of Nutrition, Food Quality and Safety, Faculty of Food Technology and Biotechnology, University of Zagreb, Pierottijeva 6, 10000 Zagreb, Croatia; ivana.rumbak@pbf.unizg.hr (I.R.); andreagradiska@gmail.com (A.G.); 2 Centre for Food Safety, Croatian Agency for Agriculture and Food, Ivana Gundulića 36b, 31000 Osijek, Croatia; martina.pavlic@hapih.hr (M.P.); darja.sokolic@hapih.hr (D.S.); 3 Department of Food and Nutrition Research, Faculty of Food Technology Osijek, Josip Juraj Strossmayer University of Osijek, Franje Kuhača 18, 31000 Osijek, Croatia; lidija.soher@ptfos.hr (L.Š.); daniela.kenjeric@ptfos.hr (D.Č.K.); 4 Department of Environmental and Occupational Health and Sports Medicine, Andrija Štampar School of Public Health, School of Medicine, University of Zagreb, Rockefeller St. 4, 10000 Zagreb, Croatia; jasna.pucarin@snz.hr; 5 Division for Environmental Health, Croatian Institute of Public Health, Rockefeller St. 7, 10000 Zagreb, Croatia

**Keywords:** dietary supplements, pediatric population, national survey, Croatia, infants, toddlers, children, adolescents, EU Menu

## Abstract

**Highlights:**

**What are the main findings?**

- The unweighted prevalence of dietary supplement use in the Croatian pediatric population was age-specific, ranging from 74.5% in infants to 13.2% in adolescents.
- Factors associated with supplement use differed between age groups and most commonly included region of residence, weight status, energy intake, physical activity level, type of diet, and presence of chronic disease

**What are the implications of the main findings?**

- The findings support age-tailored monitoring and counseling on dietary supplement use in pediatric populations.

**Abstract:**

Background/Objectives: Although the use of dietary supplements is increasing worldwide among the pediatric population, comprehensive national data on consumption patterns in Croatia remain limited. The aim of this study was to assess the prevalence and types of dietary supplement use in a national pediatric sample in Croatia, as well as the intake of active compounds and the factors influencing supplement use. Methods: This cross-sectional study analyzed data from 2078 participants from two Croatian national food consumption studies (NIPNAD 2017–2021 and NIPNOD 2018–2023), both of which followed standardized EU Menu methodology protocols. Results: The unweighted overall prevalence of dietary supplement use based on dietary records and 24 h recalls was 37.5%, highest among infants (74.5%) and declining significantly by adolescence (13.2%). Vitamin-only formulations, predominantly vitamin D, were most common in infants, whereas mixed formulations combining herbs with vitamins and minerals were most prevalent in children. Median daily vitamin and mineral intakes from dietary supplements were generally below the tolerable upper intake levels proposed by the European Food Safety Authority, except for some occasional acute exceedances in the infant group, which do not reflect habitual intake or indicate a potential risk. Several age-specific determinants of dietary supplement use emerged, including region of residence, weight status, energy intake, physical activity level, type of diet, and presence of chronic disease. Conclusions: The results of this study highlight age-specific differences in dietary supplement use driven by diverse sociodemographic and lifestyle factors. To accurately assess the risks associated with dietary supplement use, future studies must consider nutrient intake from both foods and supplements. These findings highlight the need for targeted public health programs to monitor dietary supplement use and related behaviors and to promote safe supplementation in pediatric populations.

## 1. Introduction

Within the European Union, dietary supplements are defined as foodstuffs that are concentrated sources of nutrients or other substances with a nutritional or physiological effect, presented alone or in combination with clearly indicated dosages on the product label [1]. There are different forms of dietary supplements such as capsules, pastilles, tablets, pills and other similar forms, sachets of powder, ampoules of liquids, drop dispensing bottles, and other similar forms of liquids and powders, and for each of them the European Food Safety Authority has recommended how to quantify supplement units for dietary assessment [1,2]. Although dietary supplements were designed to support a normal diet or reduce nutritional deficiencies, the prevalence of their use is increasing in Western countries [1,3]. Within Europe, the prevalence of dietary supplement use varies between 6% and 80% [4,5,6,7,8,9,10,11,12,13]. According to studies conducted outside Europe, the prevalence of dietary supplement use among children and adolescents is 20.1% to 23.5% in Australia (NHS 2014–2015), 38% in the United States (NHANES 2007–2018), 20.3% in South Korea (KNHANES 2015–2017), and 33.5% in Iran (CASPIAN-V Study) [14,15,16,17]. These variations are mostly related to the age of the studied population, with younger children tending to have a higher prevalence of dietary supplement use. Additionally, the use of different methodologies to assess dietary supplement use may influence the overall results. The two most common differences are in the dietary assessment methods used (24 h recalls, dietary records, or food propensity questionnaires—FPQs) and in the definition of supplement users, whether based only on vitamin and mineral supplements or on any type of supplement.

Despite the widespread use of dietary supplements among children and adolescents, routine supplementation is not recommended by health authorities for healthy pediatric populations. The only notable exception is the supplementation of 400 IU of vitamin D in infancy, as proposed by the ESPGHAN Committee on Nutrition [18]. Therefore, the use of dietary supplements during childhood is primarily driven by parental concern about children’s growth, as well as their beliefs, attitudes, and health practices [3]. As they grow up, adolescents take responsibility for using dietary supplements, which, in addition to parental influence, is also shaped by body image concerns, sports participation, and peer and media influence [3,5,6,8]. In European countries, the amounts of vitamins and minerals must be labeled on dietary supplement declarations [1], as independent use of dietary supplements without adequate knowledge, support, and monitoring can result in excessive micronutrient intake, which may cause adverse health outcomes [4]. Furthermore, several sociodemographic and lifestyle factors may influence dietary supplement use among infants, children, and adolescents. The use of dietary supplements decreases as children age, is lower among boys, and is less common among children and adolescents from lower-income households and those with lower parental education. Additionally, children and adolescents with overweight or obesity use dietary supplements less frequently, while those with other chronic diseases use them more often. Regular participation in physical activity is associated with greater use of dietary supplements [5,6,7,14,15,16,17].

Although dietary supplement use among various pediatric age groups has been observed in many European countries, data on their use in the Croatian population are limited. To the authors’ knowledge, only one study has reported dietary supplement use among adolescents in the city of Zagreb [19], while data on use in other pediatric age groups have not been presented. This study aimed to provide a comprehensive overview of dietary supplement use in the pediatric population in Croatia by assessing the prevalence and specific categories of supplement use. Additionally, the study aimed to estimate users’ intake of vitamins, minerals, and other active compounds from dietary supplements, and to compare their vitamin and mineral intake from supplements with the European Food Safety Authority’s dietary reference values.

## 2. Materials and Methods

### 2.1. Data Source and Study Design

Data for this cross-sectional study were obtained from 2078 participants: 1820 (aged 3 months to 9 years) from the National Food Consumption Survey on Infants and Children (NIPNAD 2017–2021) [20], and 258 (aged 10 to under 18 years) from the National Food Consumption Survey on Adolescents and Adults (NIPNOD 2018–2023) [21]. Both national studies were project-funded by the European Food Safety Authority’s “What’s on the Menu in Europe” (NIPNAD 2017–2021: OC/EFSA/DATA/2016/02 CT3; NIPNOD 2018–2023: OC/EFSA/DATA/2017/01) and follow the EU Menu methodology [22]. As a sampling frame for each population group, data from the Croatian Bureau of Statistics were used [23,24], as provided in detail in the reports [20,21]. Therefore, within each of the six regional strata, participants were randomly selected according to age and sex [20,21]. To ensure a national sample of infants, toddlers, children, and adolescents, participants were recruited using a stratified random sampling strategy based on a registry from the Ministry of Internal Affairs. The exclusion criteria were hospitalization, living in collective households and living abroad. The plausibility of energy intake was estimated, except in infants, as recommended in the EU Menu methodology [22,25,26], and participants with energy misreporting were identified but not excluded from the data analysis. The parents of participants who agreed to the study protocols signed informed consent forms and participated voluntarily. They retained the right to withdraw from the study at any point. For data analysis, participants were divided into 4 age groups according to the EFSA methodology—infants (3 months to <1 year), toddlers (1 to <3 years), children (3 to 9 years) and adolescents (10 to <18 years) [22].

Both studies were conducted in compliance with the ethical principles of the Declaration of Helsinki and were formally approved by the Ethics Committee of the Institute for Medical Research and Occupational Health (NIPNAD 2017–2021: Reg. No. 100-21/18-9, date: 20 September 2018; NIPNOD 2018–2023: Reg. No. 100-21/20-18; date 23 September 2020).

### 2.2. Study Variables and Data Collection

Data on sociodemographic characteristics and lifestyle habits were collected through a general questionnaire administered during the first face-to-face or online interview with participants’ parents or caregivers [20,21]. To address the study aims, our analysis included variables on sex (male or female), age, region (Dalmatia; Istria, Primorje and Gorski Kotar; Lika and Banovina; Northern Croatia; Slavonia; Zagreb), type of diet (normal and other dietary patterns including vegetarian, slimming, gluten-free, diabetic, and elimination diets for food allergies, as well as unspecified diets and diets for unspecified health conditions), presence of chronic diseases (yes, no), education level of interviewees (low—ISCED 0–2; medium—ISCED 3–4; high—ISCED 5–8), and employment status of interviewees (working for pay or profit, student, unemployed, maternity leave, domestic, retirement). Household income data were presented and analyzed by age group because each study defined income ranges differently. Additionally, for infants, data on birth weight and feeding patterns (breastfeeding, formula feeding, or mixed feeding) were reported.

The level of physical activity was not assessed in infants and toddlers, whereas for children and adolescents, different assessment methodologies were used, as proposed by the EU Menu methodology. Data on physical activity level (low, medium, high) for children were parent-reported [20], while for adolescents they were estimated using the International Physical Activity Questionnaire [21,27,28]. Due to these age-specific methodological differences, direct comparability between age groups was limited, and physical activity was evaluated as an age-specific determinant.

Parents reported body weight and length/height for infants, toddlers and children as measured by a pediatrician within two weeks before or after the interviews [20]. Anthropometric measurements of adolescents included stature (±0.1 cm) and body weight (±0.1 kg), assessed using a combined portable stadiometer with digital scale (Seca, type 217-type 877, Vogel & Halke GmbH & Co., Hamburg, Germany) [21]. Trained interviewers performed the measurements according to standard protocols [29]. Standardized z-scores for body mass index adjusted for sex and age were computed using AnthroPlus software version 1.0.4., based on WHO growth charts and cutoffs [30,31]. Birth weight was reported by parents. Accordingly, the infants were then divided into three groups (<2500 g, 2500–4000 g, >4000 g).

Daily energy intake was determined by averaging two non-consecutive days of dietary records (DR) for infants, toddlers, and children, and two non-consecutive 24 h recalls (24HRs) for adolescents. To capture potential fluctuations in dietary intake, a predefined sampling algorithm was used to ensure an even distribution of dietary assessments across weekdays and throughout the year. Both DR and 24HR were processed using NutriCro^®^ software (versions 2.0 and 3.0), which integrates national food composition tables with the Danish Food Database [32,33]. Energy and nutrient values were obtained from the nutrition labels of food products and beverages specific to the Croatian market. The software versions differed in the mode of administration and dietary assessment method (proxy-administered DR for infants, toddlers, and children, and interviewer-administered 24HR for adolescents), as well as in the number of added food items, products for adolescent and adult consumption, and meal recipes. If the weight of the food and beverages was not known, portion size was determined using picture books [34,35,36], predefined household measures, or the portion size indicated on the food packaging. Additionally, for infants and toddlers who were breastfed at the time of the national survey, breastfeeding frequency was recorded and breast milk intake was calculated using the methods of Paul et al. (1988) [37].

### 2.3. Assessment of Dietary Supplement Use

Dietary supplement use was assessed from the two non-consecutive DR or 24HR, depending on the population [20,21]. For each dietary supplement, data on brand, type, concentration and consumed amounts were collected. Dietary supplements were classified according to the FoodEx2 system [2], and 14 different groups of dietary supplements were identified (vitamin only; mineral only; combination of vitamin and mineral only; mixed supplements/formulations—herbal, mineral and/or vitamins; formulations containing special fatty acids; protein and amino acids; bee-produced formulations; herbal formulations and plant extracts; probiotic or prebiotic formulations; algae-based formulations—Spirulina, chlorella; fiber supplements; yeast-based formulations; enzyme-based formulations; enteral formulations). To estimate the intake of active compounds from dietary supplements, the amounts of each active compound (nutritive and non-nutrient compound) per unit listed on the dietary supplement label were used. The intakes of active compounds from dietary supplements were calculated for consumers and expressed as the average value over two days. The observed intakes of vitamins and minerals from dietary supplements were compared with European Food Safety Authority dietary reference values (EFSA DRVs) based on the age and sex [38]. Additionally, the number of children who exceeded the tolerable upper intake level (UL) from EFSA DRVs was estimated. For individual probiotic strains, only their use (yes/no) can be detected, not the amount consumed, because most probiotics specify the Colony Forming Units for the entire dietary supplement rather than for each strain. Also, only consumption (yes/no) was recorded for herbal extracts, oils, powders, and bee products because the product labels lacked information on the quantities of active compounds.

### 2.4. Statistical Analysis

Data processing and statistical analysis were carried out using IBM SPSS (version 23.0; IBM Corp., Armonk, NY, USA) and Microsoft Excel 2016 (version 16.0.5413.1000; Microsoft Corporation, Redmond, WA, USA). The agreement between the FPQ and the DR/24HR in identifying dietary supplement users was assessed using Cohen’s kappa coefficient (Table S1). Based on the level of agreement (72.9%, (χ^2^ (1, *N* = 2078) = 614.418, κ = 0.486, *p* < 0.001), the DR/24HR was used as the primary data source for identifying dietary supplement users and assessing intake of active compounds.

Descriptive statistics were used to estimate the prevalence of dietary supplement use and active compound intake as unweighted sample values. Descriptive data were presented for the total pediatric population across the four defined age groups. Certain sociodemographic, anthropometric, and lifestyle factors were age-specific and were analyzed only within their corresponding pediatric subgroups. The analysis of active compound intake from dietary supplements and its contribution to reference values for vitamins and minerals was conducted among users of each compound. Statistical significance in all tests was defined as *p* < 0.05.

Given the non-normal distribution indicated by the Shapiro–Wilk test, continuous variables were reported as medians with interquartile ranges. For active compounds with ≤2 supplement users identified, only the median value was presented. Categorical data were presented as frequencies (*n*) or proportions (%). To compare the prevalence of dietary supplement use among pediatric age groups, chi-square tests or Fisher’s exact tests were performed, depending on the observed frequencies in the contingency tables. Differences among age groups in the intake of active compounds and the contribution of vitamin and mineral intake to EFSA DRV values were analyzed using the Mann–Whitney U test or the Kruskal–Wallis test, followed by Dunn’s post hoc test for comparisons between age groups with adjusted significance levels using Bonferroni for multiple comparisons. Age-specific comparisons of sociodemographic, lifestyle, and anthropometric characteristics between supplement users and non-users were conducted using the Mann–Whitney U test (continuous variables) and the chi-square test (categorical variables).

Multivariate binary logistic regression was used to identify determinants of dietary supplement use, with results presented as adjusted odds ratios with 95% confidence intervals. Analyses were performed separately for each age group. The potential covariates were first evaluated using crude binary logistic regression models (Table S14). Before constructing the final multivariate models, selected sociodemographic and lifestyle variables with low proportions of missing data (infants: two variables with 0.9% and 2%; children: one variable with 0.5%) were imputed using the mode for categorical variables and the median for continuous variables. Household income was the variable with the most missing data (infants and toddlers: 7%; children: 9%; adolescents: 1.5%) and was excluded from the multivariate analysis. A backward elimination method was applied to optimize and stabilize the models; covariates showing multicollinearity or excessively large standard errors due to low frequencies within age groups were excluded. In the final models, variables were included as follows: for infants, z-score for BMI-for-age (continuous), region (Zagreb as reference), and infant feeding habits (breastfeeding as reference); for toddlers, energy intake (continuous), z-score for BMI-for-age (continuous), dietary habits (normal dietary patterns as reference), and chronic diseases (no as reference); for children, region (Zagreb as reference), physical activity level (low as reference), and dietary habits (normal dietary patterns as reference); for adolescents, z-score for BMI-for-age (continuous), dietary habits (normal dietary patterns as reference), chronic diseases (no as reference), and physical activity level (low as reference). Post hoc sensitivity analysis of sociodemographic, lifestyle, and anthropometric characteristics as factors associated with the use of dietary supplements was conducted as case analysis without imputations (Figure S1), which confirmed that missing data did not alter the regression outcomes.

Finally, post hoc sensitivity analysis was conducted including plausible reporters and presented in Table S2.

## 3. Results

### 3.1. Dietary Supplement Use

In the total pediatric sample in Croatia (*N* = 2078), based on DR and 24HR, the overall prevalence of dietary supplement use was 37.5% (Table 1). According to the FPQ (Table S1), a higher prevalence of 61.2% was observed; however, cross-classification of participants by both methods showed an overall agreement of 72.9% (χ^2^(1, *N* = 2078) = 614.418, κ = 0.486, *p* < 0.001). An unweighted prevalence of dietary supplement use based on DR and 24HR was the highest in infants (74.5%), decreasing with age (*p* < 0.001); in adolescents, 13.2% were dietary supplement users. Infants used a significantly lower number of dietary supplements per participant (*p* = 0.002) compared to other age groups.

In the total sample, unweighted estimates based on DR and 24 h recalls showed that the most frequently used dietary supplements were vitamin-only formulations (22.6%) and mixed formulations containing herbal compounds, minerals, and/or vitamins (11.4%). The prevalence of dietary supplement users taking only vitamins, minerals, or probiotics decreased from infancy to adolescence. A significantly higher prevalence of combinations of vitamin and mineral supplements, mixed formulations, formulations containing special fatty acids, and bee-produced formulations was observed among toddlers and children. The prevalence of protein and amino acid supplement users was highest among adolescents (3.2%).

### 3.2. Estimation of Active Compound Intake

**Figure 1:**
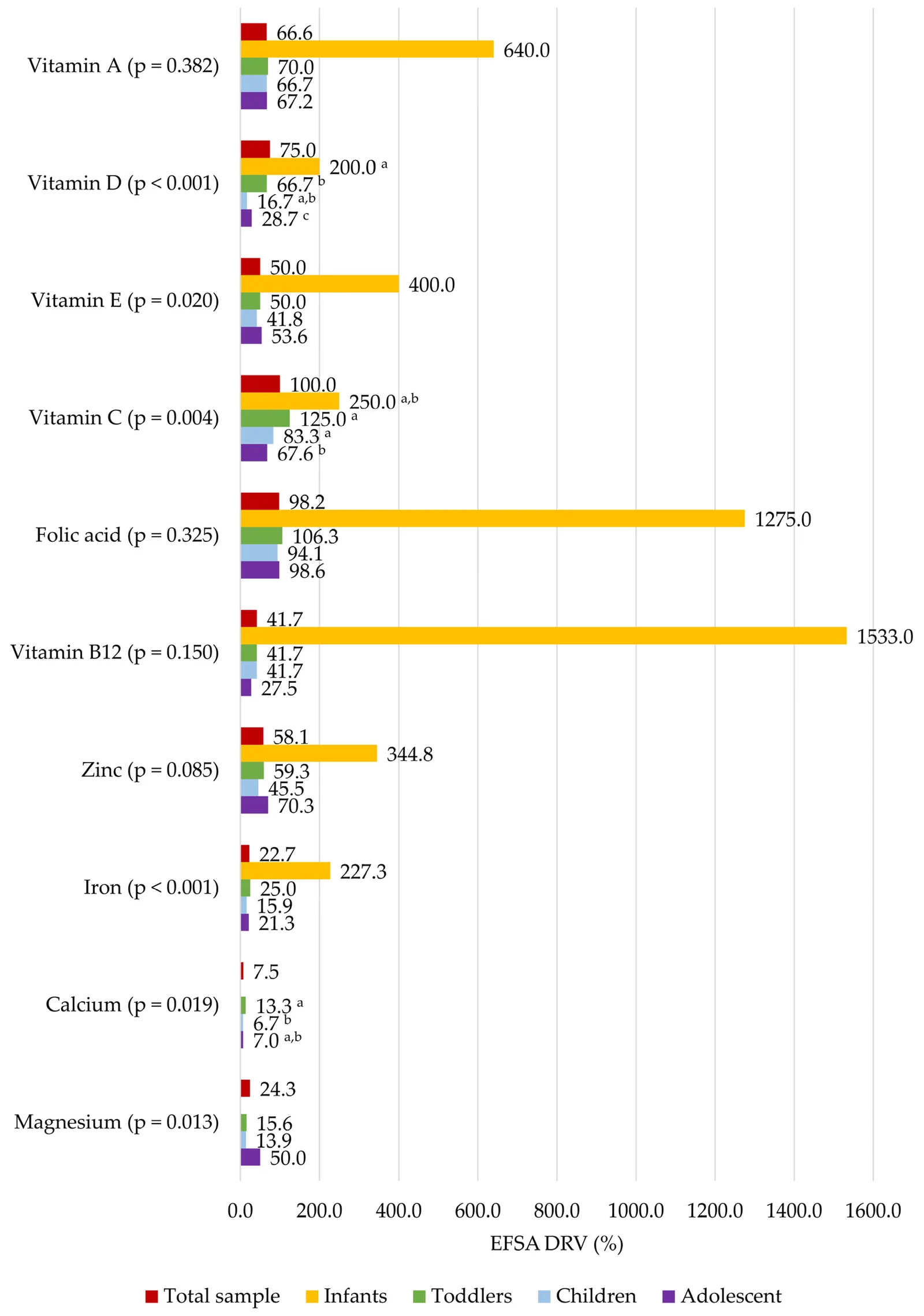
Contribution of selected vitamin and mineral intake from dietary supplements to EFSA DRVs among supplement users in the Croatian pediatric population participating in the NIPNAD 2017–2021 and NIPNOD 2018–2023 surveys. Significant differences (*p* < 0.05) across age groups were assessed using the Kruskal–Wallis test with post hoc Dunn’s test (^a–c^ denote group differences).

The unweighted prevalence and median daily intakes of selected nutrients from dietary supplements estimated from the DR and 24HR are presented in Table 2 and Table 3, while their contributions to recommended daily intake according to the EFSA DRVs are shown in Figure 1. Detailed results are provided in the Supplementary Materials (Tables S3–S13; Figures S2 and S3). For macronutrients, we highlight the use of protein supplements, docosahexaenoic acid (DHA), and eicosapentaenoic acid (EPA). The prevalence of protein supplement use was observed among adolescents (2.7%). The prevalence of DHA and EPA supplement use was slightly below 2% in the overall pediatric population and was lowest among adolescents (0.8%; *p* = 0.001 and *p* = 0.006, respectively). However, no significant differences in daily intakes were observed between age groups.

Among the vitamins, results are presented for the use of vitamins D, E, A, and C, folate, and vitamin B12. Vitamin D was the most commonly used dietary supplement (27.7%), and its use differed significantly between groups. The prevalence of vitamin D use was highest in infants (71.7%) and then decreased significantly (*p* < 0.001) with age, to 35.7% in toddlers, 14.5% in children, and 4.5% in adolescents. Accordingly, daily vitamin D intake from dietary supplements decreased significantly (*p* = 0.032) from 20 µg per day in infants to 4.3 µg per day in adolescents, reflecting changes in the recommended daily vitamin D intake according to EFSA DRVs. In total, 14 participants (0.7%) had a median vitamin D intake from dietary supplements above the UL of EFSA DRVs. Vitamin C was the second most commonly used vitamin (14.0%) from dietary supplements. Its use was high in toddlers (17.6%) and children (18.6%), and low among infants (1.2%) and adolescents (5.4%). Despite this, no difference was found in the daily median vitamin C intake from dietary supplements between age groups, while its contribution to recommended daily intake according to EFSA DRVs decreased from infancy to adolescence (*p* = 0.004). Vitamin E use was most prevalent among toddlers and children and significantly less common among infants and adolescents (*p* < 0.001). Median vitamin E intake from dietary supplements did not differ between age groups; however, its contribution to the recommended daily intake relative to the EFSA DRVs was significantly higher in toddlers than in other age groups (*p* = 0.020). No participant exceeded the UL for vitamin E according to the EFSA DRVs through dietary supplements. Vitamin A was used by 6.8% of the total pediatric population, and its prevalence trend across age groups was similar to that of vitamin E supplement use. Adolescents had the highest median daily intake of vitamin A from dietary supplements (400 µg RE/day; *p* = 0.032), while the contribution to the recommended daily intake relative to the EFSA DRVs was highest in infants because of lower recommended values. The UL according to EFSA DRVs for vitamin A from dietary supplements was exceeded by six participants (0.3%) in the total sample. Folic acid and vitamin B_12_ were used most often (both *p* < 0.001) among toddlers (6.5% and 12.0%) and children (7.7% and 13.2%) compared with adolescents and infants. The highest daily intake of folic acid and vitamin B_12_ from dietary supplements was observed in infants, but only for one participant, who had high acute use. Based on the established UL, 14 participants (0.7%) exceeded it for folic acid intake from dietary supplements. It should be noted that estimated intakes of several vitamins (vitamin A, vitamin E, folic acid, and vitamin B12) were based on a very small number of participants (*n* ≤ 2) in the infant population and may not reflect typical intake patterns in the broader infant population. Therefore, these results should be interpreted with caution as descriptive values.

The prevalence of mineral use showed that the most commonly used dietary supplements were zinc (7.1%), iron (6.4%), iodine (5.3%), and calcium (4.3%). Minerals with a moderate prevalence of use (1–2%) included magnesium, selenium, manganese, copper, and chromium, while the remaining minerals had a prevalence of less than 1%. We focused on supplementation with zinc, iron, calcium, and magnesium because of their established public health relevance in these population groups. In Croatia, mandatory salt iodization is the primary national public health strategy for preventing iodine deficiency. There is no consistent pattern of higher mineral intake from dietary supplements across specific age groups; rather, prevalence depends on the particular mineral. The highest prevalence of zinc and calcium intake (both *p* < 0.001) from dietary supplements was observed among toddlers and children, while the highest prevalence of iron intake was observed among infants (9.0% and 5.4%) and children (9.6% and 5.5%). In contrast, the highest prevalence (*p* < 0.001) of magnesium use was observed in adolescents (4.3%). Median daily intakes of zinc and calcium from dietary supplements did not differ between age groups, and these intakes corresponded to about 58.1% of the EFSA DRVs for zinc and 7.5% for calcium. Infants had the highest median daily iron intake from dietary supplements at 50.0 mg per day (*p* < 0.001), while other age groups consumed less than 3 mg per day. With these intakes from dietary supplements, infants exceeded 200% of the EFSA DRVs, a significant difference (*p* < 0.001) compared with other age groups. The ULs from the EFSA DRVs were exceeded for zinc from dietary supplements in seven participants (0.3%). However, the extreme zinc intake values in the infant subgroup were estimated from a very small number of participants (*n* ≤ 2) and should therefore be interpreted with caution and considered only descriptive. Adolescents had a significantly higher estimated daily intake of magnesium (125.0 mg/day; *p* = 0.004) from dietary supplements.

### 3.3. Sociodemographic, Anthropometric, and Lifestyle Characteristics as Factors Associated with the Use of Dietary Supplements

Descriptive statistics for sociodemographic, anthropometric, and lifestyle characteristics, along with tested differences between dietary supplement users and non-users within each age group, are presented in Table 4. No differences in dietary supplement use by sex were found in any age group. Region has been shown to be a significant factor in dietary supplement consumption among infants (*p* < 0.001) and children (*p* = 0.013), with the highest proportion of consumers observed in Zagreb and the lowest proportion in Lika and Banovina region. Higher parental education was significantly more common among dietary supplement users than among non-users in the overall pediatric population (79.9% vs. 70.8%, *p* < 0.001), but not within each age group. Furthermore, infants from families with higher household incomes were more likely to use dietary supplements (65.0% vs. 48.8%, *p* = 0.026), but this trend was not observed in other age groups. Dietary supplement use did not differ by parental employment status. Of the lifestyle habits, significantly more toddlers (15.4% vs. 6.6%, *p* = 0.001) and adolescents (26.5% vs. 9.8%, *p* = 0.005) who had chronic diseases used dietary supplements. Infant feeding habits were not related to dietary supplement use, but type of diet was related to dietary supplement use in adolescents. Significantly more adolescents who practiced specific diets (vegetarian, slimming, gluten-free, diabetic, elimination diets for food allergies, unspecified diets, and diets for unspecified health conditions) used dietary supplements than those with normal dietary patterns (20.6% vs. 5.4%, *p* = 0.005). Among toddlers, dietary supplement users had a lower median daily energy intake (1041 (849–1234) kcal/day vs. 1109 (931–1275) kcal/day, *p* = 0.005), whereas among adolescents, supplement users had a higher median daily energy intake (1925 (1642–2283) kcal/day vs. 1737 (1493–2048) kcal/day, *p* = 0.036). Physical activity level was significantly related to dietary supplement use in children (in high physical activity category—users: 45.3%; non-users: 35.7%; *p* = 0.023), but not in adolescents. Analysis of anthropometric characteristics indicates that, in the total population, the BMI-for-age z-score was significantly lower (*p* < 0.001) in dietary supplement users, and that more participants who were underweight or had adequate nutritional status used dietary supplements (*p* = 0.008). There was no difference between birth weight among infants.

**Figure 2:**
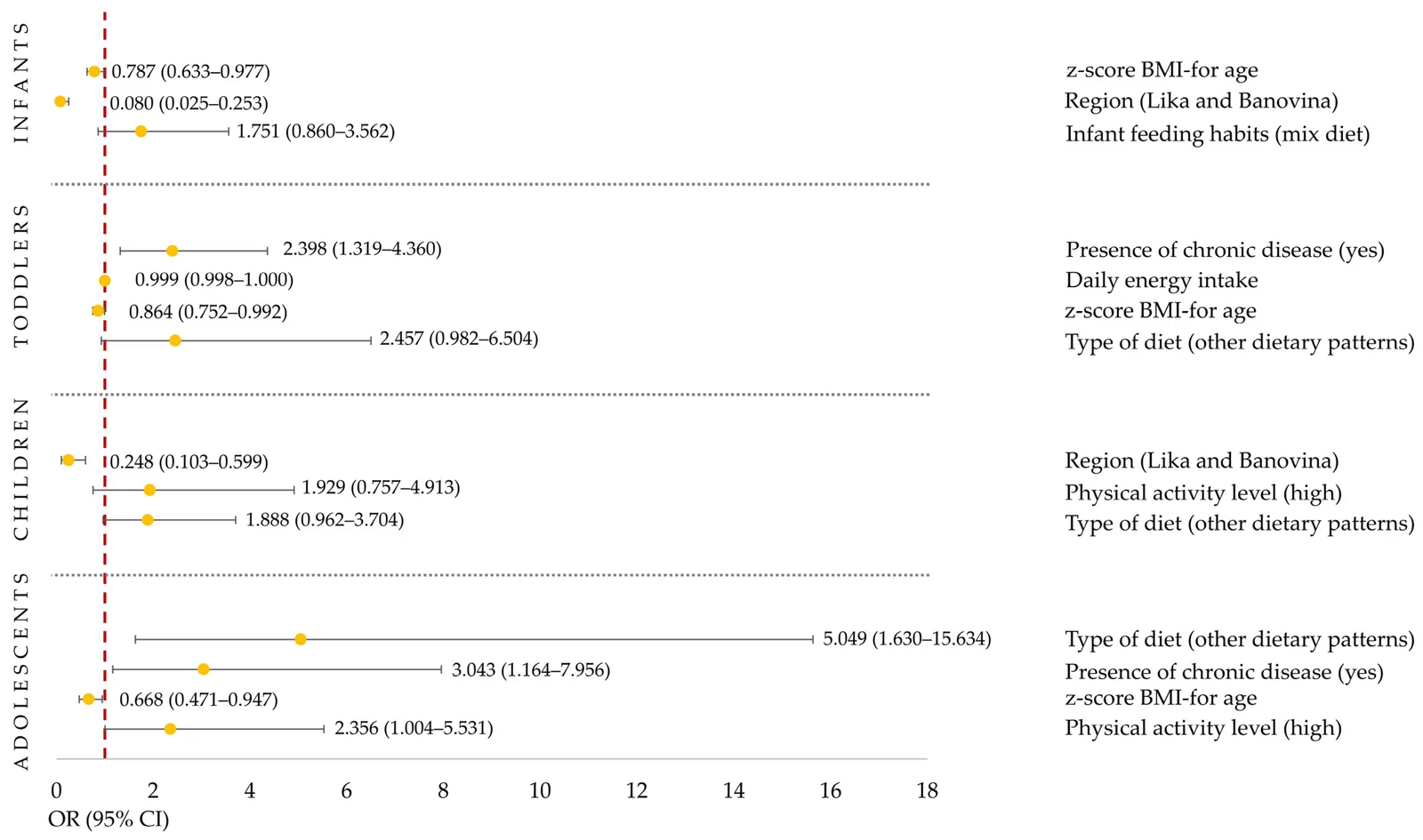
Sociodemographic, lifestyle and anthropometric characteristics as factors associated with use of dietary supplements in Croatian pediatric population participating in the NIPNAD 2017–2021 and NIPNOD 2018–2023 surveys. Odds ratios with corresponding 95% confidence intervals from multivariate binary logistic regression models.

The significant variables identified were included in the multivariate binary logistic regression models shown in Figure 2. Variables that caused model instability or were not significant in the crude binary logistic regression analysis (Table S14) were excluded. In infants, BMI-for-age z-score and region of residence were significant determinates of dietary supplement use. An increase in BMI-for-age z-score reduced the likelihood of use dietary supplements (OR: 0.787; 95%CI: 0.633–0.977; *p* = 0.030). Regarding region of residence, infants living in Lika and Banovina region had lower odds of dietary supplement use compared with those from Zagreb (OR: 0.080; 95%CI: 0.025–0.253; *p* < 0.001). Although infant feeding patterns were not statistically significant, feeding with a combination of breastfeeding and infant formula showed a trend toward higher intake of dietary supplements. In toddlers, greater odds of dietary supplement use were associated with lower BMI-for-age z-scores (OR: 0.864; 95%CI: 0.752–0.992; *p* = 0.039) and reduced daily energy intake (OR: 0.999; 95%CI: 0.998–1.000; *p* = 0.006). One of the strongest determinates in this model was the presence of chronic disease (OR: 2.398; 95%CI: 1.319–4.360; *p* = 0.004). The marginal statistical significance of diet type suggests a trend toward increased dietary supplement use among toddlers who did not have typical dietary patterns. 

In children, those living in Lika and Banovina were less likely to use dietary supplement compared to children living in Zagreb (OR: 0.249; 95%CI: 0.103–0.599; *p* = 0.002). Although physical activity level was significant in the overall model (*p* = 0.017), significance was not observed when each physical activity level category was analyzed. As in toddlers, the marginal statistical significance of diet type suggests a trend toward increased dietary supplement use among children who did not follow typical dietary patterns. During adolescence, diet type is the strongest determinant of dietary supplement use, with a fivefold higher likelihood of using dietary supplements among those who did not follow typical dietary patterns (OR: 5.049; 95%CI: 1.630–15.634; *p* = 0.005). In addition, the presence of chronic disease (OR: 3.043; 95%CI: 1.164–7.956, *p* = 0.023) and lower BMI-for-age z-scores were associated with higher odds of dietary supplement use (OR: 0.668; 95%CI: 0.471–0.947, *p* = 0.023). A high level of physical activity was associated with an increased likelihood of dietary supplement use (OR: 2.356; 95%CI: 1.004–5.531, *p* = 0.049).

## 4. Discussion

The study provides insight into dietary supplement use patterns among the pediatric population in Croatia. Based on DR and 24HR the overall unweighted prevalence of dietary supplement use was 37.5%, with significant differences between age groups. Infants had the highest prevalence at 74.5%, followed by toddlers (46.2%), children (26.9%), and adolescents (13.2%). In European countries, the use of dietary supplements in this population greatly varies, with higher prevalence (>50%) reported in Scandinavian countries [4,11] and prevalence ranges from 15 to 40% in others [6,7,8,9,10,12,13,39]. Consistent with our findings, existing literature indicates a decline in dietary supplement use from childhood to adolescence [7,9,11,12,39,40]. Additionally, data from the French population show significantly higher use of dietary supplements in infants and toddlers aged 0 to 15 months (47.2%) compared with other pediatric age groups (14.4–19.9%) [13]. The lack of age-specific differences in dietary supplement use was reported in several previous studies, potentially reflecting narrow subgroup distributions within pediatric populations, such as Danish participants aged 4 to 10 years [4], and German and Slovenian participants aged 12 to 17 years [6,8]. In Croatia, one study on dietary supplement use has been conducted, but only among adolescents. It estimated that about 17.2% of adolescents aged 15–16 years and 20.9% of those aged 17–18 years use dietary supplements, which is slightly higher than in the current study for adolescents [19]. Differences in the prevalence of dietary supplement use between countries may arise for several reasons, primarily the age of participants and how they are divided into subgroups. Our study showed that supplement intake varies by age, which can affect the overall picture of dietary supplement use in pediatric populations. Furthermore, there is a considerable variation in the methods used to assess dietary supplement use. The most common approaches were food frequency questionnaires or food propensity questionnaires assessing supplement use over the past months or year, multiple 24 h recalls and dietary records, and interviews with questions about supplement use during the past week or month. In addition, the grouping and classification of dietary supplements vary between studies, as do the definitions of dietary supplement users: some studies classify them as users of any dietary supplements, while others include only those who use only vitamin and mineral formulations. These issues are well known [41], and by harmonizing the methodology for food consumption data, EFSA is trying to minimize this problem [2,22]. In national Croatian food consumption studies, both approaches are used: for infants, toddlers, and children, dietary records (DR) and a food propensity questionnaire (FPQ) are applied, and for adolescents, 24 h recalls (24HR) and an FPQ are used. For the present study, data from DR and 24HR were used to identify dietary supplement users, as these methods provide more detailed insight into the type, amount, and dosage of supplements used. Although they primarily capture short-term dietary supplement use and do not assess habitual use as the FPQ does, this methodological approach was chosen because the agreement test between DR/24HR and FPQ showed a significantly moderate association (Table S1).

Among the Croatian pediatric population, the most commonly used supplements were vitamin-only preparations, followed by mixed formulations and probiotics. Vitamin and probiotic use were highest in infants and toddlers, while mixed formulations were used mainly in toddlers and children. In Croatia, pediatricians routinely supplement infants and toddlers with vitamin D, which may contribute to higher overall use of vitamin-type dietary supplements, particularly vitamin D as the active compound in dietary supplements. Vitamin D was used by 27.7% of the total study population, including 71.1% of infants and 35.7% of toddlers. In pediatric populations, vitamins are generally used more often than minerals or other non-nutrient active compounds in dietary supplements [6,7,10,11,12,39,40]. Moreover, vitamin D is one of the most frequently utilized active compounds among dietary supplement users, along with vitamins C, A, and E [6,7,11,12,40]. In the overall Croatian pediatric population, toddlers, children, and adolescents used minerals as active compounds in dietary supplements more often than infants. Across the total sample and all age groups, zinc, iron, iodine, and calcium were the minerals most commonly used in dietary supplements, consistent with European trends in mineral intake from supplements [6,7,12]. In addition, a higher prevalence of magnesium intake from dietary supplements was found among adolescents, which is also consistent with other studies [7,12]. In early childhood, parents choose dietary supplements for their children, while adolescents have autonomy in choosing their own supplements. Parents most often select dietary supplements they believe support children’s growth and development and protect against flu and infections, whereas adolescents choose dietary supplements that they believe enhance athletic performance, increase muscle mass, improve concentration, and are associated with an ideal body type [3]. The use of vitamin and mineral dietary supplements influences total daily nutrient intake. In this study, we assessed the contribution of vitamins and minerals from dietary supplements to recommended total daily intake, expressed as a percentage of EFSA DRVs by age and sex. Vitamin intake from dietary supplements averaged 40–70% of the EFSA DRVs, except for pantothenic acid and vitamin B12, which were around 40%, and folic acid and vitamin K, which were closer to 100%. A smaller contribution, <40% of the EFSA DRVs, was observed for mineral intake from dietary supplements, except for manganese, molybdenum, selenium, and zinc, which had higher contributions to the DRVs. Only a few studies examine how vitamin and mineral intake from dietary supplements contributes to overall daily intake or recommendations [4,19,42]. Moreover, these studies indicate a growing need for parental education about dietary supplementation in the pediatric population because dietary supplement use can exceed the recommended intake of certain vitamins (e.g., vitamin A, vitamin C, some vitamins from the B-complex) and minerals (e.g., copper, zinc, and iodine). In the present study, we assessed intakes of vitamins and minerals from dietary supplements only against the EFSA DRVs, which do not provide a complete picture of how many children exceed the daily intakes of micronutrients. Therefore, these results should be interpreted with caution. Considering only dietary supplements, 14 participants had higher intakes of vitamin D and folic acid than the ULs, two participants had higher intakes of selenium and fluoride, and one participant had higher intakes of zinc and molybdenum.

In the present study, we observed a low prevalence of dietary supplements containing proteins, amino acids, amino acid-related compounds, fatty acids, lipid formulations, prebiotics, and functional carbohydrates, with pronounced age-specific intake patterns (Table S5). Protein supplements were consumed primarily by adolescents, whereas omega-3 fatty acids were more commonly used by toddlers and children. A slightly higher prevalence (4.5%) was noted for beta-glucan supplements, again particularly among toddlers and children. These results are unsurprising and consistent with previous studies. The use of protein-based dietary supplements during adolescence has been reported [5,8,10,12] and is associated with the desire to improve body image, increase muscle mass, and enhance physical performance [3]. Moreover, the intake of omega-3 fatty acids and functional carbohydrates from dietary supplements was higher in toddlers and children [11,12], as these nutrients are of particular concern to parents and caregivers for supporting neurological development and overall health, as well as strengthening immunity and reducing infections at that age [3,43,44]. Furthermore, we highlight the prevalence of the three most commonly used probiotic strains (Table S6): *Lacticaseibacillus rhamnosus* (6.2%), *Bifidobacterium lactis* (4.6%), and *Limosilactobacillus reuteri* (2.4%). In line with global trends, our results in the Croatian pediatric population reveal age-specific usage patterns of probiotic strains [3,45]. Among infants, the highest use was recorded for the *Limosilactobacillus reuteri* strain, directly reflecting clinical guidelines for minimizing infantile colic and early functional gastrointestinal symptoms [46]. In contrast, in toddlers and children, more frequent use of the *Lacticaseibacillus rhamnosus* and *Bifidobacterium animalis* subsp. *lactis* strains was observed. These strains are the primary compounds in preparations intended to support the immune system during the period when children attend group educational settings. In addition, both strains are recognized in the literature as the gold standard for the prevention and treatment of antibiotic-associated diarrhea, to which older children are significantly more often exposed than infants [3,46]. The prevalence of other non-nutrient active compounds from dietary supplements was low (less than 2%) and they were used primarily by toddlers and children (Tables S6–S13). Studies on these dietary supplement groups in the general pediatric population are rare [12]. Propolis (1.3%) and honey (1.7%) are the most commonly used bee products, with no use in infants, reflecting parents’ clinical caution due to the risk of infant botulism [47]. Among herbal extracts, elderberry (1.7%) and rosehip (0.6%) are most common, with markedly higher use in children (2.7% and 1.1%, respectively). The observed intake of non-nutrient compounds from dietary supplements in this study raises an important public health concern, particularly because there are no established recommended intake values or maximum safe levels.

Lastly, the results of this research indicate that place of residence, weight status, energy intake, physical activity level, type of diet, and presence of chronic disease are the most prominent characteristics of the Croatian pediatric population that may affect dietary supplement use. Moreover, the results reveal age-specific interactions among these factors. These factors have been shown in most studies to be significant determinants of dietary supplement use [4,5,6,7,8,39]. In this study, place of residence refers to the region and was a significant factor for infants and children. Infants and children living in the Lika and Banovina region were less likely to use dietary supplements. This finding may be due to the smaller proportion of the pediatric population in this region and, consequently, the lower prevalence of dietary supplement use. Additionally, given the predominantly rural characteristics of this region, the results may reflect underlying contextual factors such as socioeconomic inequalities, lower household incomes, and reduced geographic accessibility to specialist pediatric care and pharmacy networks compared with Zagreb. However, because these contextual factors were not directly measured in the present study, this hypothesis should be further assessed in population-based studies using socioecological models. In Germany, by contrast, place of residence was not a significant determinant of dietary supplement use [6], and similarly, there is no difference in the prevalence of dietary supplement use between children and adolescents living in urban and rural areas of Turkey [39]. Weight status was a significant factor in all three groups, with children who had a higher BMI-for-age z-score showing a lower likelihood of dietary supplement use. This trend is also observed in other countries, where obese or overweight children have lower odds of using dietary supplements than children with adequate body weight [6,7,39]. Parents are more likely to give dietary supplements to children who are undernourished or have adequate body weight because of concerns about growth and development [3], even though children with obesity may also have nutritional depletion, and supplementation is one component of multicomponent treatment interventions [48]. The presence of chronic diseases and deviations from normal dietary patterns can increase parental concern about the need for dietary supplements [3,7]. Thus, in toddlers and adolescents, chronic conditions were among the main factors driving the use of dietary supplements in the Croatian pediatric population. Additionally, adolescents who followed a different dietary pattern than usual, such as vegetarian, slimming, gluten-free, diabetic, and elimination diets for food allergies, as well as unspecified diets and diets for unspecified health conditions, were significantly more likely to use dietary supplements, whereas the influence of diet on dietary supplement use showed a non-significant trend in toddlers and children. Increased physical activity is regularly accompanied by more frequent use of dietary supplements to enhance sports performance [49]. Therefore, it is not surprising that as physical activity levels rise or exercise is performed more often, children and adolescents use dietary supplements more frequently. Consistent with the existing literature [5,6,7,8], this study showed that only during adolescence were participants with higher levels of physical activity more likely to use dietary supplements. In the present study, sex was not identified as a significant factor in dietary supplement use in any age group, suggesting that parental decisions and adolescent behavior are driven by factors other than sex. Several studies have also failed to find an association between sex and dietary supplement use [4,39]. Differences have been observed in dietary supplement use among adolescents, but the results remain inconclusive as to whether girls or boys are less likely to use them [5,6,7]. Additionally, parental education level, employment status, and household income did not appear to influence dietary supplement use in the Croatian pediatric population. These results may be due to the low frequency of dietary supplement use within specific pediatric age groups when these factors are taken into account. As with sex, there is no consensus on the influence of these parameters on dietary supplement use [4,6,7,39]. Specifically, in Denmark and Germany, these factors have not been shown to determine dietary supplement use [4,6], whereas in Poland and Turkey, children of parents with higher levels of education, higher household income, and parents who work in healthcare are more likely to use dietary supplements [7,39]. It is important to emphasize that birth weight was not a determinant of dietary supplement use in our population, whereas in Korea, lower birth weight is associated with more frequent dietary supplement use, particularly vitamin and mineral supplements and vitamin D supplements [16]. Furthermore, among Korean children, the influence of infant feeding habits on overall dietary supplement use was not evident. However, omega-3 fatty acid/fish oil supplement use was more prevalent in formula-fed children, and zinc supplement use was more prevalent in breastfed children [16]. In the present study, we did not find a significant association with infant feeding habits, but we observed a trend indicating that infants who were mixed breastfed and formula-fed had higher odds of using dietary supplements.

This is the first study to map dietary supplementation in a pediatric population in Croatia. EU methodology was used for data collection and analysis, ensuring high-quality data. One of the limitations is the cross-sectional design of the study, which does not allow causal inferences. In accordance with the EFSA EU Menu methodology, energy intake misreporting was calculated for toddlers, children, and adolescents. To assess the potential impact of energy misreporting on dietary supplement use, a sensitivity analysis was performed (Table S2). The prevalence of supplement use was slightly lower among misreporters (who represented 8.9% of the overall sample) than among plausible reporters, with no differences across the supplement categories. Moreover, the prevalence among plausible reporters in each age group was similar to that observed in the total study sample, with the same differences between them. Therefore, we retained participants with misreported energy intake in the analysis to preserve sample representativeness. To identify supplement users, DR/24HRwere used. Because these methods focus on short-term intake, they may underestimate habitual or intermittent supplement use, potentially introducing bias. However, this assessment approach provided precise information on supplement brands and quantities, enabling a comprehensive analysis of dietary supplement intake. Furthermore, cross-classification of the DR/24HR assessment with the FPQ demonstrated moderate agreement (κ = 0.486, *p* < 0.001), indicating that DR/24HR adequately represent overall supplementation patterns. Reliance on DR/24HR to assess intake of active compounds from dietary supplements provides only short-term, acute intake data rather than habitual, long-term intake. Consequently, the proportion of participants who exceed the UL should be interpreted with caution as reflecting acute intake on the recorded survey days, which does not confirm chronic overexposure in the population. Additionally, data on brands and concentrations depended on parents’ memory and the product declarations; therefore, some active compounds could not be accurately quantified, so these active compounds were analyzed as intake frequencies. Micronutrient intake was assessed only from dietary supplements, but estimating total intake from both supplements and food sources would provide a more accurate evaluation of overall intake and allow identification of participants who exceed the UL. Further studies should combine micronutrient intakes from both sources and use statistical modeling of usual intake to estimate long-term chronic risk. Another limitation that must be emphasized is that survey weights were not applied to the datasets, as specific post-stratification weights were not available for each pediatric and adolescent population. Reporting unweighted estimates is common in sub-cohort evaluations with dietary surveys to avoid bias when general population weights are not fully recalibrated for each age group. In this study, the prevalence of dietary supplement use and the regression analyses were examined across the pediatric sub-cohort. Consequently, the observed unweighted prevalence of 37.5% should not be interpreted as a definitive national prevalence, but rather as an unweighted estimate from the sample. Although the sampling protocol adhered to the EFSA EU Menu methodology to achieve a nationally representative demographic and geographic sample, the results on dietary supplement use should be interpreted with caution due to underweighted sample estimates and when generalizing the prevalence of dietary supplement use to the broader population.

## 5. Conclusions

This is the first study to map patterns of dietary supplement use in the pediatric population in Croatia. Based on dietary records and 24 h recalls, an unweighted overall prevalence of 37.5% was found, with a decline in the proportion of children using dietary supplements as age increases from infancy to adolescence. Across the total study population, vitamin supplementation, especially vitamin D, predominates. A higher prevalence of probiotic use is also notable in infants and toddlers, while use of protein supplements and combinations of vitamins, minerals, and other compounds is more often found in adolescents. Observed median daily nutrient intake from dietary supplements aligned with EFSA dietary reference values. However, occasional acute exceedances of the tolerable upper intake levels from dietary supplements have been observed. Use of various non-nutrient compounds for which no safety limits exist indicates the need to educate parents and children, especially adolescents, about the proper use of dietary supplements. Because these estimates are based on unweighted samples, the findings should be interpreted with caution and confirmed in future studies that apply habitual intake modeling. To evaluate the long-term safety of supplementation, future studies should integrate habitual supplement use with nutrient intake from food sources. The results also highlight age-specific determinants of dietary supplement use, underscoring the need for age-tailored monitoring, counseling, and public health communication.

## Figures and Tables

**Table 1:** Estimated unweighted prevalence of dietary supplement use based on dietary records and 24 h recalls among the Croatian pediatric population participating in the NIPNAD 2017–2021 and NIPNOD 2018–2023 surveys.

Nutrient	Total Sample (*N* = 2078)	Infants (*n* = 322)	Toddlers (*n* = 535)	Children (*n* = 963)	Adolescents (*n* = 258)	*p* Value *
Users (%) ^1^	37.5	74.5	46.2	26.9	13.2	<0.001
No. of supplements (*n*) ^1^	1 (1–2)	1 (1–1) ^a^	1 (1–2) ^b^	1 (1–2) ^b^	1 (1–2) ^b^	0.002
Vitamin only (%)	22.6	68.3	29.9	8.6	2.3	<0.001
Mineral only (%)	2.0	6.2	1.7	0.6	2.7	<0.001
Combination of vitamin and mineral only (%)	1.6	0.0	1.5	2.4	1.2	0.011
Mixed formulations ^2^ (%)	11.4	0.9	15.3	14.7	3.9	<0.001
Formulations containing special fatty acids (%)	1.3	0.0	2.1	1.5	1.2	0.044
Protein and amino acids (%)	0.4	0.0	0.2	0.0	3.1	<0.001
Bee-produced formulations (%)	1.7	0.0	1.3	2.6	1.2	0.004
Herbal formulations and plant extracts (%)	0.7	0.0	0.3	0.8	0.4	0.366
Probiotic or prebiotic formulations (%)	6.6	14.0	11.4	3.1	0.4	<0.001
Algae-based formulations (%)	0.1	0.0	0.0	0.1	0.4	0.403
Fiber supplements (%)	0.1	0.0	0.2	0.1	0.0	0.999
Yeast-based formulations (%)	0.3	0.6	0.4	0.2	0.0	0.497
Enzyme-based formulations (%)	0.1	0.0	0.0	0.1	0.0	0.999
Enteral formulations (%)	0.4	0.0	1.1	0.0	0.0	0.002

^1^ Any type of dietary supplements; ^2^ Included herbal, minerals and/or vitamins; * Significant differences (*p* < 0.05) across age groups were assessed using the chi-square test or Fisher’s exact test, and the Kruskal–Wallis test with post hoc Dunn’s test (^a,b^ denote group differences).

**Table 2:** Estimated unweighted prevalence of selected nutrients from dietary supplements based on dietary records and 24 h recalls among the Croatian pediatric population participating in the NIPNAD 2017–2021 and NIPNOD 2018–2023 surveys.

Nutrient	Total Sample (*N* = 2078)	Infants (*n* = 322)	Toddlers (*n* = 535)	Children (*n* = 963)	Adolescents (*n* = 258)	*p* Value *
Macronutrients:						
Proteins (%)	0.3	0.0	0.0	0.0	2.7	<0.001
Docosahexaenoic acid (%)	1.9	0.6	3.9	1.6	0.8	0.001
Eicosapentaenoic acid (%)	1.4	0.0	2.8	1.3	0.8	0.006
Vitamins:						
Vitamin A (%)	6.8	0.3	8.2	9.1	3.5	<0.001
Vitamin D (%)	27.7	71.7	35.7	14.5	5.4	<0.001
Vitamin E (%)	8.9	0.3	10.8	11.8	4.7	<0.001
Vitamin C (%)	14.0	1.2	17.6	18.6	5.4	<0.001
Folic acid (%)	5.8	0.3	6.5	7.7	3.9	<0.001
Vitamin B_12_ (%)	9.8	0.3	12.0	13.2	4.7	<0.001
Minerals:						
Zinc (%)	7.1	0.3	9.0	9.6	2.7	<0.001
Iron (%)	6.4	6.2	7.9	6.5	3.1	0.085
Calcium (%)	4.3	0.0	5.4	5.5	3.1	<0.001
Magnesium (%)	1.3	0.0	1.5	0.7	4.3	<0.001

* Significant differences (*p* < 0.05) across age groups were assessed using the chi-square test or Fisher’s exact test.

**Table 3:** Estimated daily nutrient intake based on dietary records and 24 h recalls for selected nutrients from dietary supplements among supplement users in the Croatian pediatric population participating in the NIPNAD 2017–2021 and NIPNOD 2018–2023 surveys.

Nutrient	Total Sample	Infants	Toddlers	Children	Adolescents	*p* Value *
Macronutrients						
Proteins (g)	23.0 (12.3–23.5)	-	-	-	23.0 (12.3–23.5)	NC
Docosahexaenoic acid (mg)	117.5 (25.2–450.0)	15.0 (10.0–20.0)	125.0 (25.3–600.0)	300.0 (75.0–450.0)	44.5 (34.0–55.0)	0.191
Eicosapentaenoic acid (mg)	200.0 (25.0–400.0)	-	200.0 (21.9–400.0)	200.0 (165.0–400.0)	44.3 (6.0–82.5)	0.325
Vitamins						
Vitamin A (µg RE)	200.0 (100.0–300.0)	1600.0 (N/A)	175.0 (100.0–225.0)	200.0 (100.0–350.0)	400.0 (200.0–540.0)	0.032
Vitamin D (µg)	10.0 5.0–20.0)	20.0 (10.0–20.0) ^a^	10.0 (9.7–20.0) ^a^	2.5 (2.5–10.0) ^a,b^	4.3 (2.5–6.0) ^b^	<0.001
Vitamin E (mg α-TE)	4.0 (2.5–6.0)	20.0 (N/A)	3.0 (2.1–6.0)	3.8 (3.0–6.0)	6.4 (3.5–9.4)	0.080
Vitamin C (mg)	25.0 (10.0–50.0)	50.0 (37.5–115.0)	25.0 (10.0–50.0)	25.0 (10.0–51.2)	42.0 (15.0–50.0)	0.393
Folic acid (µg)	100.0 (45.0–100.0)	600 (N/A)	75.0 (25.0–100.0)	100.0 (50.0–100.0)	116.1 (100.0–200.0)	0.034
Vitamin B_12_ (µg)	0.6 (0.3–1.3)	23.0 (N/A)	0.6 (0.3–1.3)	0.6 (0.3–1.3)	1.0 (0.4–1.4)	0.160
Minerals						
Zinc (mg)	2.5 (1.3–4.4)	10.0 (N/A)	2.6 (1.3–4.0)	2.5 (1.3–4.0)	5.2 (3.8–9.0)	0.068
Iron (mg)	1.8 (0.9–7.0)	50.0 (25.0–50.0) ^a^	1.8 (0.9–7.0) ^b^	1.5 (0.9–1.8) ^b^	2.6 (1.3–4.7) ^b^	<0.001
Calcium (mg)	60.0 (30.0–60.0)	-	60.0 (30.0–60.0)	32.0 (30.0–60.0)	80.0 (45.0–200.0)	0.059
Magnesium (mg)	51.3 (25.5–125.0)	-	26.5 (13.1–39.0) ^a^	32.0 (18.8–72.9) ^a,b^	125.0 (69.3–168.8) ^b^	0.004

Results are presented as median (interquartile range); “-“ no intake; N/A—the interquartile range was not calculated for subgroups with very small sample sizes (*n* ≤ 2), and these estimates should be considered descriptive and interpreted with caution; NC—*p* Value was not calculated as consumption was identified in only one subgroup; * Significant differences (*p* < 0.05) across age groups were assessed using the Kruskal–Wallis test with post hoc Dunn’s test (^a,b^ denote group differences).

**Table 4:** Differences in sociodemographic, lifestyle and anthropometric characteristics among dietary supplement users and non-users of Croatian pediatric population participating in the NIPNAD 2017–2021 and NIPNOD 2018–2023 surveys.

Parameters	Total Sample (*N* = 2078)			Infants (*n* = 322)			Toddlers (*n* = 535)			Children (*n* = 963)			Adolescents (*n* = 258)		
	NDS	DS	*p* Value *	NDS	DS	*p* Value *	NDS	DS	*p* Value *	NDS	DS	*p* Value *	NDS	DS	*p* Value *
Sex (%):															
Boys	50.8	54.0	0.157	46.3	56.7	0.105	52.4	50.2	0.607	51.0	54.8	0.291	49.6	55.9	0.492
Girls	49.2	46.0		53.7	43.3		47.6	49.8		49.0	45.2		50.4	44.1	
Region (%):															
Dalmatia	19.3	18.3	<0.001	18.3	14.9	<0.001	20.5	17.4	0.303	19.6	22.8	0.013	17.4	23.5	0.355
Istra, Primorje and Gorski Kotar	11.3	11.5		12.2	10.6		13.5	10.9		10.2	12.7		11.6	17.6	
Lika and Banovina	8.5	2.3		17.1	5.9		5.2	2.8		8.7	2.3		8.9	0.0	
Northern Croatia	14.9	11.3		7.3	9.6		9.0	10.1		14.8	12.4		25.9	17.6	
Slavonia	17.5	20.0		18.3	23.9		18.4	17.0		18.3	18.1		13.4	14.7	
Zagreb	28.4	36.5		26.8	35.1		33.3	41.7		28.4	31.7		22.8	26.5	
Person completing the questionnaires ^1^ (%):															
Mother	94.5	97.1	0.026	93.9	98.3	0.036	97.6	98.0	0.752	96.0	96.9	0.519	86.2	82.4	0.771
Father	4.9	2.7		6.1	1.7		2.4	2.0		4.0	3.1		10.3	11.8	
Adolescent	0.6	0.3		-	-		-	-		-	-		3.6	5.9	
Parents education level ^2^ (%):															
Low (ISCED 0–2)	1.3	0.4	<0.001	0.0	0.4	0.164	0.3	0.0	0.337	0.4	0.0	0.088	5.8	5.9	0.998
Medium (ISCED 3–4)	27.9	19.7		29.3	19.6		19.1	15.4		27.3	21.2		40.6	41.2	
High (ISCED 5–8)	70.8	79.9		70.0	80.0		80.6	84.6		72.3	78.8		536	52.9	
Parents employment status (%):															
Employed for pay or profit	84.2	87.1	0.072	72.0	85.0	0.120	84.4	87.9	0.569	86.1	88.4	0.789	82.6	85.3	0.479
Student	1.1	1.0		1.2	0.8		0.7	0.8		0.6	0.4		3.1	8.8	
Unemployed	11.2	9.7		22.0	11.3		11.5	9.7		10.4	8.9		9.8	5.9	
Maternity leave or sick	1.4	1.7		3.7	2.5		1.7	1.2		1.1	1.5		0.9	0.0	
Housewife	1.8	0.5		1.2	0.4		1.7	0.4		1.7	0.8		2.2	0.0	
Retired	0.3	0.0		0.0	0.0		0.0	0.0		0.1	0.0		1.3	0.0	
Household income NIPNAD 2017–2021 ^3^ (%):															
<378 €	0.4	0.3	<0.001	2.5	0.0	0.026	0.0	0.9	0.174	0.3	0.0	0.094	-	-	N/A ^8^
378–757 €	3.9	2.2		6.3	3.7		4.5	1.7		3.5	1.3		-	-	
758–1141 €	10.9	11.2		17.5	13.8		7.1	9.1		11.8	10.8		-	-	
1142–1513 €	25.4	20.4		25.0	17.5		26.2	23.5		25.2	20.0		-	-	
>1513 €	59.2	65.9		48.8	65.0		62.2	64.8		59.3	67.9		-	-	
Household income NIPNOD 2018–2023 ^3^ (%):															
<451 €	1.4	0.0	0.179	-	-	N/A	-	-	N/A	-	-	N/A	1.4	0.0	0.179
451–902 €	4.5	8.8		-	-		-	-		-	-		4.5	8.8	
903–1354 €	23.2	8.8		-	-		-	-		-	-		23.2	8.8	
1355–1805 €	70.9	82.4		-	-		-	-		-	-		70.9	82.4	
Presence of chronic disease (%):															
No	90.5	87.1	0.016	95.1	90.4	0.184	93.6	84.6	0.001	88.9	88.7	0.699	90.2	73.5	0.005
Yes	9.5	12.9		4.9	9.6		6.6	15.4		11.1	11.3		9.8	26.5	
Physical activity level ^4^ (%):															
Low	13.8	6.2	<0.001	-	-	N/A	-	-	N/A	3.1	2.3	0.023	47.3	35.3	0.107
Medium	52.1	48.3		-	-		-	-		61.2	52.3		23.7	17.6	
High	34.1	45.5		-	-		-	-		35.7	45.3		29.0	47.1	
Type of diet (%):															
Normal diet	96.8	92.9	<0.001	98.8	93.8	0.345	97.9	93.1	0.056	96.9	96.1	0.226	94.6	79.4	0.005
Other dietary patterns ^5^	3.2	7.1		1.2	6.2		2.1	6.9		3.1	3.9		5.4	20.6	
Infants feeding habits ^6^ (%):															
Breastfeeding	47.6	42.6	0.356	47.6	42.6	0.356	-	-	N/A	-	-	N/A	-	-	N/A
Formula use	34.1	31.2		34.1	31.2		-	-		-	-		-	-	
Mix diet	18.3	26.6		18.3	26.2		-	-		-	-		-	-	
Energy intake (kcal), M (IQR) ^7^	1353 (1102–1664)	1105 (867–1389)	<0.001	814 (693–1022)	876 (732–1046)	0.160	1109 (931–1275)	1041 (849–1234)	0.005	1425 (1196–1678)	1392 (1188–1706)	0.493	1737 (1493–2048)	1925 (1642–2283)	0.036
Body weight (kg), M (IQR)	20.7 (14.0–32.0)	12.5 (9.5–19.0)	<0.001	8.5 (7.5–9.5)	8.3 (7.5–9.5)	0.431	13.0 (11.5–14.0)	12.0 (10.6–13.5)	<0.001	23.0 (18.0–28.8)	21.0 (17.5–26.0)	0.002	54.0 (47.0–65.0)	51.5 (44.0–57.0)	0.067
Body length/height (cm), M (IQR)	117.0 (96.0–137.0)	88.0 (74.2–113.0)	<0.001	71.0 (68.0–74.0)	71.0 (68.0–74.0)	0.982	88.8 (83.0–95.0)	86.0 (81.0–91.8)	<0.001	122.0 (110.0–132.0)	118.0 (107.0–128.0)	0.002	165.0 (156.8–173.0)	162.2 (157.0–172.0)	0.438
Body mass index (kg/m^2^), M (IQR)	16.4 (15.0–18.2)	16.2 (14.9–17.5)	0.002	17.0 (15.9–18.3)	16.5 (15.6–17.8)	0.111	16.2 (15.2–17.5)	16.2 (15.0–17.3)	0.333	15.6 (14.5–17.1)	15.5 (14.1–16.9)	0.209	20.0 (18.2–21.3)	18.0 (17.3–21.3)	0.030
z-score of body mass index for age, M (IQR)	0.2 (−0.6–1.0)	0.0 (−0.9–0.8)	<0.001	0.1 (−0.9–0.9)	−0.3 (−1.1–0.5)	0.050	0.3 (−0.5–1.1)	0.2 (−0.6–0.9)	0.105	0.1 (−0.7–0.9)	0.0 (−1.0–0.9)	0.445	0.3 (−0.5–1.1)	−0.1 (−1.0–0.9)	0.117
Category of z-score of body mass index for age (%):															
Underweight	4.1	5.8	0.008	3.7	7.1	0.156	4.5	4.9	0.497	4.7	5.4	0.766	1.8	5.9	0.440
Normal weight	71.1	74.5		72.0	77.9		69.4	73.3		71.1	72.6		71.0	73.5	
At risk/Overweight	17.2	15.1		18.3	12.5		18.1	17.0		15.1	15.4		22.3	17.6	
Obese	7.6	4.6		6.1	2.5		8.0	4.9		8.5	6.6		4.9	2.9	
Birth weight ^6^ (%):															
<2500 g	1.2	3.4	0.508	1.2	3.4	0.508	-	-	N/A	-	-	N/A	-	-	N/A
2500–4000 g	87.7	83.3		87.7	83.3		-	-		-	-		-	-	
>4000 g	11.1	13.2		11.1	13.2		-	-		-	-		-	-	

NDS–non dietary supplement users; DS–dietary supplement users; ^1^ In the NIPNAD–2017–2021 survey, the questionnaire could be completed only by a proxy respondent; ^2^ ISCED–the International Standard Classification of Education; ^3^ During study official currency in Croatia was Croatian Kuna (1 EUR = 7.53450 HRK); ^4^ Surveyed in children and adolescents; ^5^ Includes vegetarian, slimming, gluten-free, diabetic, and elimination diets for food allergies, as well as unspecified diets and diets for unspecified health conditions. ^6^ Surveyed in infants; ^7^ Median (interquartile range); ^8^ N/A—not applicable; - no data; * Age groups were compared using a chi-squared test or Fisher exact test, and the Mann–Whitney U test (*p* < 0.05).

## Data Availability

The data are available from the Croatian Agency for Agriculture and Food, but restrictions apply to the availability of these data. Data are available upon request and with permission of the Croatian Agency for Agriculture and Food.

## Supplementary Materials

The following supporting information can be downloaded at: https://www.mdpi.com/article/10.3390/children13101434/s1, Table S1: Analysis of agreement and correlation between 24 h recalls/dietary records and food propensity questionnaire for detecting dietary supplement users; Table S2: Post hoc analysis of the plausibility of reports of prevalence of dietary supplement use among the Croatian pediatric population participating in the NIPNAD 2017–2021 and NIPNOD 2018–2023; Table S3: Unweighted prevalence of vitamin use and daily vitamin intake from different types of dietary supplements among supplement users in the Croatian pediatric population participating in the NIPNAD 2017–2021 and NIPNOD 2018–2023 surveys; Table S4: Unweighted prevalence of mineral use and daily mineral intake from dietary supplements among supplement users in the Croatian pediatric population participating in the NIPNAD 2017–2021 and NIPNOD 2018–2023 surveys; Table S5: Unweighted prevalence of use and daily intake of active compounds from supplements containing proteins, amino acids, amino acid-related compounds, fatty acids, lipid formulations, prebiotics and functional carbohydrates among dietary supplement users in the Croatian pediatric population participating in the NIPNAD 2017–2021 and NIPNOD 2018–2023 surveys; Table S6: Unweighted prevalence of probiotic species use among the Croatian pediatric population participating in the NIPNAD 2017–2021 and NIPNOD 2018–2023 surveys; Table S7: Unweighted prevalence of bee-produce formulations use among the Croatian pediatric population participating in the NIPNAD 2017–2021 and NIPNOD 2018–2023 surveys; Table S8: Unweighted prevalence of digestive enzymes use among the Croatian pediatric population participating in the NIPNAD 2017–2021 and NIPNOD 2018–2023 surveys; Table S9: Unweighted prevalence of phytochemicals formulation use among the Croatian pediatric population participating in the NIPNAD 2017–2021 and NIPNOD 2018–2023 surveys; Table S10: Unweighted prevalence of other active compound use among the Croatian pediatric population participating in the NIPNAD 2017–2021 and NIPNOD 2018–2023 surveys; Table S11: Unweighted prevalence of essential oil use among the Croatian pediatric population participating in the NIPNAD 2017–2021 and NIPNOD 2018–2023 surveys; Table S12: Unweighted prevalence of plant powders, concentrates and whole-food preparations use among the Croatian pediatric population participating in the NIPNAD 2017–2021 and NIPNOD 2018–2023 surveys; Table S13: Unweighted prevalence of plant extracts use among the Croatian pediatric population participating in the NIPNAD 2017–2021 and NIPNOD 2018–2023 surveys; Table S14: Crude binary logistic regression of sociodemographic, lifestyle and anthropometric characteristics as factors associated with use of dietary supplements in Croatian pediatric population participating in the NIPNAD 2017–2021 and NIPNOD 2018–2023 surveys; Figure S1: Sensitivity analysis of sociodemographic, lifestyle and anthropometric characteristics as factors associated with use of dietary supplements in Croatian pediatric population participating in the NIPNAD 2017–2021 and NIPNOD 2018–2023 surveys; Figure S2: Contribution of vitamins from dietary supplements to EFSA DRVs among supplement users in the Croatian pediatric population participating in the NIPNAD 2017–2021 and NIPNOD 2018–2023 surveys; Figure S3: Contribution of minerals from dietary supplements to EFSA DRVs among supplement users in the Croatian pediatric population participating in the NIPNAD 2017–2021 and NIPNOD 2018–2023 surveys.

## Abbreviations

The following abbreviations are used in this manuscript:

BMI	Body Mass Index
DHA	Docosahexaenoic acid
DRV	Dietary Reference Values
DR	Dietary records
DS	Dietary supplement users
EFSA	European Food Safety Authority
EPA	Eicosapentaenoic acid
FPQ	Food Propensity Questionnaire
ISCED	International Standard Classification of Education
NDS	Non dietary supplement users
NIPNAD 2017–2021	National Food Consumption Survey on Infants and Children
NIPNOD 2018–2023	National Food Consumption Survey on Adolescents and Adults
UL	Tolerable Upper Intake Level
