# Supplementary material for: Dietary Supplement Use from Infancy to Adolescence in Croatia: Prevalence, Types and Associated Factors
Source: Children (Basel). 2026 Oct 19;13(10):1434. doi: 10.3390/children13101434 (PMC13703828; doi:10.3390/children13101434)
Supplement: Supplementary file 1 [file children-13-01434-s001.zip › children-4514687-supplementary.pdf]

SUPPLEMENTARY MATERIALS

Table S1. Analysis of agreement and correlation between 24-hour recalls/dietary records and food propensity questionnaire for detecting dietary supplement users 1

Table S2. Post hoc analysis of the plausibility of reports of prevalence of dietary supplement use among the Croatian pediatric population participating in the NIPNAD 2017–2021 and NIPNOD 2018–2023 2

Figure S1. Sensitivity analysis of sociodemographic, lifestyle and anthropometric characteristics as factors associated with use of dietary supplements in Croatian pediatric population participating in the NIPNAD 2017–2021 and NIPNOD 2018–2023 surveys. 3

Table S3. Unweighted prevalence of vitamin use and daily vitamin intake from different types of dietary supplements among supplement users in the Croatian pediatric population participating in the NIPNAD 2017–2021 and NIPNOD 2018–2023 surveys. 4

Figure S2. Contribution of vitamins from dietary supplements to EFSA DRVs among supplement users in the Croatian pediatric population participating in the NIPNAD 2017–2021 and NIPNOD 2018–2023 surveys.. 6

Table S4. Unweighted prevalence of mineral use and daily mineral intake from dietary supplements among supplement users in the Croatian pediatric population participating in the NIPNAD 2017–2021 and NIPNOD 2018–2023 surveys. 7

Figure S3. Contribution of minerals from dietary supplements to EFSA DRVs among supplement users in the Croatian pediatric population participating in the NIPNAD 2017–2021 and NIPNOD 2018–2023 surveys.. 9

Table S5. Unweighted prevalence of use and daily intake of active compounds from supplements containing proteins, amino acids, amino acid-related compounds, fatty acids, lipid formulations, prebiotics and functional carbohydrates among dietary supplement users in the Croatian pediatric population participating in the NIPNAD 2017–2021 and NIPNOD 2018–2023 surveys. 10

Table S6. Unweighted prevalence of probiotic species use among the Croatian pediatric population participating in the NIPNAD 2017–2021 and NIPNOD 2018–2023 surveys. 12

Table S7. Unweighted prevalence of bee-produce formulations use among the Croatian pediatric population participating in the NIPNAD 2017–2021 and NIPNOD 2018–2023 surveys 13

Table S8. Unweighted prevalence of digestive enzymes use among the Croatian pediatric population participating in the NIPNAD 2017–2021 and NIPNOD 2018–2023 surveys 13

Table S9. Unweighted prevalence of phytochemicals formulation use among the Croatian pediatric population participating in the NIPNAD 2017–2021 and NIPNOD 2018–2023 surveys 13

Table S10. Unweighted prevalence of other active compound use among the Croatian pediatric population participating in the NIPNAD 2017–2021 and NIPNOD 2018–2023 surveys 14

Table S11. Unweighted prevalence of essential oil use among the Croatian pediatric population participating in the NIPNAD 2017–2021 and NIPNOD 2018–2023 surveys 14

Table S12. Unweighted prevalence of plant powders, concentrates and whole-food preparations use among the Croatian pediatric population participating in the NIPNAD 2017–2021 and NIPNOD 2018–2023 surveys 14

Table S13. Unweighted prevalence of plant extracts use among the Croatian pediatric population participating in the NIPNAD 2017–2021 and NIPNOD 2018–2023 surveys 15

Table S14. Crude binary logistic regression of sociodemographic, lifestyle and anthropometric characteristics as factors associated with use of dietary supplements in Croatian pediatric population participating in the NIPNAD 2017–2021 and NIPNOD 2018–2023 surveys. 16

Table S1. Analysis of agreement and correlation between 24-hour recalls/dietary records and food propensity questionnaire for detecting dietary supplement users

Variables	24hr/DR (non-users)	24hr/DR (users)	Total	χ^2 (df = 1)	Cohen's κ
**FPQ (non-users)**	770 (37.1 %)	36 (1.7%)	806 (38.8%)		
**FPQ (users)**	528 (25.4%)	744 (35.8%)	1272 (61.2%)		
**Total**	1298 (62.5%)	780 (37.5%)	2078 (100.0%)	614.148 *	0.486 *

Variables are presented as n (%); 24hr – 24-hour recalls; DR – dietary records; FPQ – food propensity questionnaire; * $p < 0.001$

To test the agreement and connection between the 24-hour recalls/dietary records and the food propensity questionnaire, a contingency table analysis was performed with calculation of the chi-square test and Cohen's K coefficient (Table S1). The overall percentage agreement between the two variables is 72.9%, with mutual non-users detected in 37.1% of cases and mutual dietary supplement users detected in 35.8% of cases. The results of the chi-square test indicate a highly statistically significant relationship between the analyzed variables (χ^2 (1, N = 2078) = 614.418; $p <$ 0.001). The value of Cohen's κ coefficient is κ = 0.486 ($p < 0.001$), which indicates a statistically significant and moderate agreement between the methods.

Table S2. Post hoc analysis of the plausibility of reports of prevalence of dietary supplement use among the Croatian pediatric population participating in the NIPNAD 2017–2021 and NIPNOD 2018–2023

Dietary supplement	Total study sample [1]			Total plausible reports			
	Misreports (n = 158)	Plausible reports (n = 1598)	p Value *	Toddlers (n = 506)	Children (n = 918)	Adolescents (n = 174)	*p* Value *
Users (%) [2]	23.4	31.5	0.036	46.0	26.6	14.9	< 0.001
Vitamin only (%)	9.5	14.6	0.077	29.4	8.7	2.9	< 0.001
Mineral only (%)	1.3	1.3	0.988	1.8	0.5	3.4	0.003
Combination of vitamin and mineral only (%)	0.6	2.1	0.213	1.6	2.5	1.1	0.433
Mixed formulations [3] (%)	11.4	13.5	0.454	15.2	14.2	5.2	0.003
Formulations containing special fatty acids (%)	0.6	1.7	0.312	2.2	1.4	1.7	0.555
Protein and amino acids (%)	2.5	0.3	0.006	0.2	0.0	2.3	< 0.001
Bee-produced formulations (%)	2.5	1.9	0.612	1.4	2.4	1.1	0.381
Herbal formulations and plant extracts (%)	0.0	0.9	0.238	1.0	0.9	0.6	0.925
Probiotic or prebiotic formulations (%)	2.5	5.5	0.109	11.3	3.3	0.6	< 0.001
Algae based formulations (%)	0.6	0.1	0.172	0.0	0.0	0.6	0.109
Fiber supplements (%)	0.0	0.1	0.656	0.2	0.1	0.0	0.799
Yeast based formulations (%)	0.0	0.3	0.529	0.4	0.2	0.0	0.760
Enzyme-based formulations (%)	0.0	0.1	0.753	0.0	0.1	0.0	0.690
Enteral formulations (%)	0.6	0.4	0.530	1.2	0.0	0.6	0.002

[1] Plausibility assessment was not performed for infants; [2] Any type of dietary supplements; [3] Included herbals, minerals and/or vitamins; * Age groups were compared using a chi-squared test or Fisher exact test ($p < 0.05$).

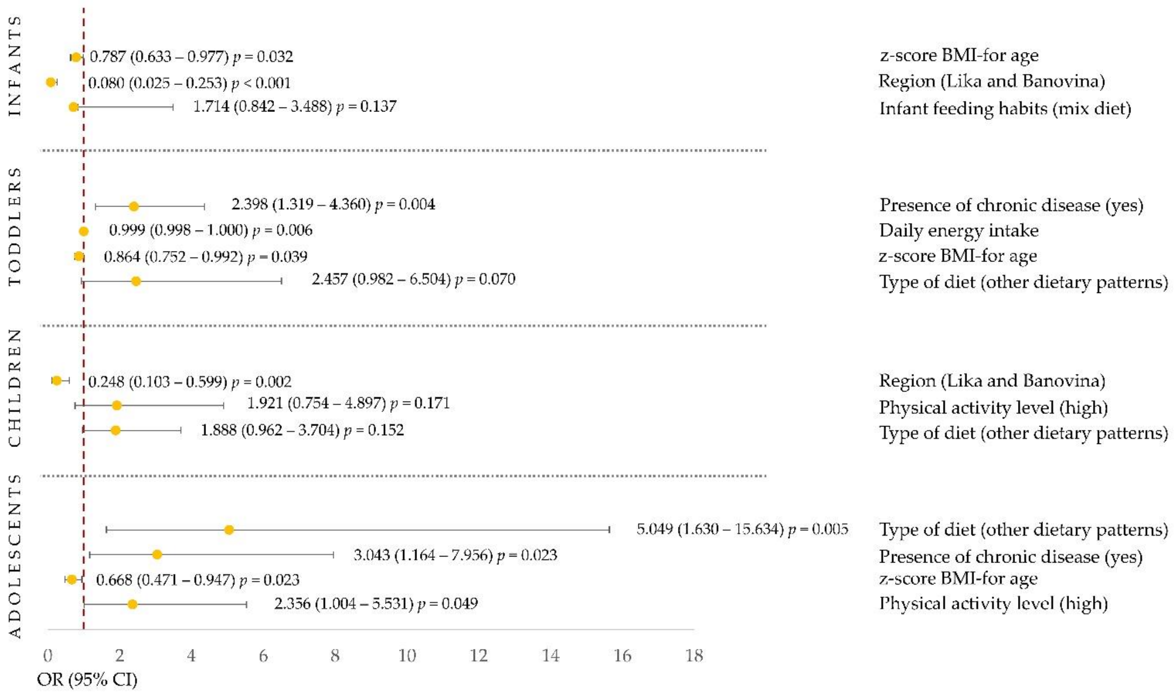

Figure S1. Sensitivity analysis of sociodemographic, lifestyle and anthropometric characteristics as factors associated with use of dietary supplements in Croatian pediatric population participating in the NIPNAD 2017–2021 and NIPNOD 2018–2023 surveys. Odds ratios with corresponding 95% confidence intervals from multivariate binary logistic regression models

Table S3. Unweighted prevalence of vitamin use and daily vitamin intake from different types of dietary supplements among supplement users in the Croatian pediatric population participating in the NIPNAD 2017–2021 and NIPNOD 2018–2023 surveys.

**Active compound**	**Total sample (N = 2078)**	**Infants (n = 322)**	**Toddlers (n = 535)**	**Children (n = 963)**	**Adolescents (n = 258)**	***p* Value ***
Vitamin A (µg RE), % [1]; M (IQR) [2]	*6.8;* 200.0 (100.0 – 300.0)	*0.3;* 1600.0 (N/A)	*8.2;* 175.0 (100.0 – 225.0)	*9.1;* 200.0 (100.0 – 350.0)	*3.5;* 400.0 (200.0 – 540.0)	*< 0.001* 0.032
Vitamin D (µg), %; M (IQR)	*27.7;* 10.0 (5.0 – 20.0)	*71.7;* 20.0 (10.0 – 20.0) [a]	*35.7;* 10.0 (9.7 – 20.0) [a]	*14.5;* 2.5 (2.5 – 10.0) [a,b]	*5.4;* 4.3 (2.5 – 6.0) [b]	*< 0.001* < 0.001
Vitamin E (mg α-TE), %; M (IQR)	*8.9;* 4.0 (2.5 – 6.0)	*0.3;* 20.0 (N/A)	*10.8;* 3.0 (2.1 – 6.0)	*11.8;* 3.8 (3.0 – 6.0)	*4.7;* 6.4 (3.5 – 9.4)	*< 0.001* 0.080
Vitamin K (µg), %; M (IQR)	*0.9;* 20.0 (11.3 – 37.0)	*0.9;* 25.0 (25.0 – 32.5) [a, b]	*1.3;* 9.0 (6.4 – 13.5) [a]	*0.6;* 40.0 (20.0 – 40.0) [b]	*1.2;* 17.0 (14.8 – 25.5) [a, b]	*0.500* 0.015
Vitamin C (mg), %; M (IQR)	*14.0;* 25.0 (10.0 – 50.0)	*1.2;* 50.0 (37.5 – 115.0)	*17.6;* 25.0 (10.0 – 50.0)	*18.6;* 25.0 (10.0 – 51.2)	*5.4;* 42.0 (15.0 – 50.0)	*< 0.001* 0.393
Thiamine (mg), %; M (IQR)	*8.4;* 0.3 (0.1 – 0.5)	*0.3;* 1.6 (N/A)	*11.2;* 0.3 (0.1 – 0.5)	*11.0;* 0.3 (0.1 – 0.5)	*4.3;* 0.8 (0.4 – 1.1)	*< 0.001* 0.029
Riboflavin (mg), %; M (IQR)	*8.6;* 0.4 (0.2 – 0.7)	*0.3;* 1.8 (N/A)	*11.2;* 0.4 (0.2 – 0.7)	*11.1;* 0.4 (0.2 – 0.7)	*4.3;* 0.8 (0.4 – 1.4)	*< 0.001* 0.053
Niacin (mg NE), %; M (IQR)	*9.2;* 4.0 (2.0 – 8.0)	*0.3;* 20.0 (N/A)	*11.0;* 4.0 (2.0 – 8.0)	*12.4;* 4.0 (2.0 – 8.0)	*4.7;* 9.9 (4.2 – 15.3)	*< 0.001* 0.029
Pantothenic acid (mg), %; M (IQR)	*8.2;* 1.5 (0.8 – 3.0)	*0.3;* 8.0 (N/A)	*10.5;* 1.5 (0.8 – 3.0)	*10.8;* 1.5 (0.8 – 3.0)	*3.9;* 2.2 (1.4 – 6.0)	*< 0.001* 0.255
Vitamin B_6 (mg), %; M (IQR)	*9.4;* 0.4 (0.2 – 0.7)	*0.3;* 1.0 (N/A) [a, b]	*11.4;* 0.4 (0.2 – 0.7) [a]	*12.6;* 0.4 (0.2 – 0.7) [a]	*5.0;* 1.1 (0.7 – 1.4) [b]	*< 0.001* 0.007
Biotin (µg), %; M (IQR)	*8.7;* 12.5 (6.3 – 20.0)	*0.3;* 30.0 (N/A) [a, b]	*10.1;* 10.0 (6.3 – 12.5) [a]	*11.8;* 12.5 (6.3 – 25.0) [a, b]	*4.3;* 25.0 (15.0 – 50.0) [b]	*< 0.001* 0.008
Folic acid (µg), %; M (IQR)	*5.8;* 100.0 (45.0 – 100.0)	*0.3;* 600 (N/A)	*6.5;* 75.0 (25.0 – 100.0)	*7.7;* 100.0 (50.0 – 100.0)	*3.9;* 116.1 (100.0 – 200.0)	*< 0.001* 0.034

Table S3. *Continued*

Vitamin B_{12} (µg), %; M (IQR)	*9.8;*<br>0.6 (0.3 – 1.3)	*0.3;*<br>23.0 (N/A)	*12.0;*<br>0.6 (0.3 – 1.3)	*13.2;*<br>0.6 (0.3 – 1.3)	*4.7;*<br>1.0 (0.4 – 1.4)	*< 0.001*<br>0.160
Choline (mg), %; M (IQR)	*1.4;*<br>9.0 (5.0 – 31.4)	*0.0;*<br>-	*2.4;*<br>30.0 (10.0 – 33.0)	*1.6;*<br>5.0 (0.4 – 9.0)	*0.4;*<br>70.6 (N/A)	*0.006*<br>0.022
Beta-carotene (mg), %; M (IQR)	*0.5;*<br>1.6 (1.0 – 2.0)	*0.3;*<br>2.0 (N/A)	*0.2;*<br>1.0 (N/A)	*0.8;*<br>1.6 (1.0 – 2.0)	*0.0;*<br>-	*0.276*<br>0.450

[1] Percentage of vitamin users; [2] Median (interquartile range); - no intake; N/A – interquartile range was not calculated for subgroups with very small sample sizes ($n \leq 2$); * Age groups were compared using a chi-squared test or Fisher exact test (italic p Values), and the Kruskal-Wallis test with post hoc Dunn's test (adjusted significance), where a, b indicated significant differences ($p < 0.05$).

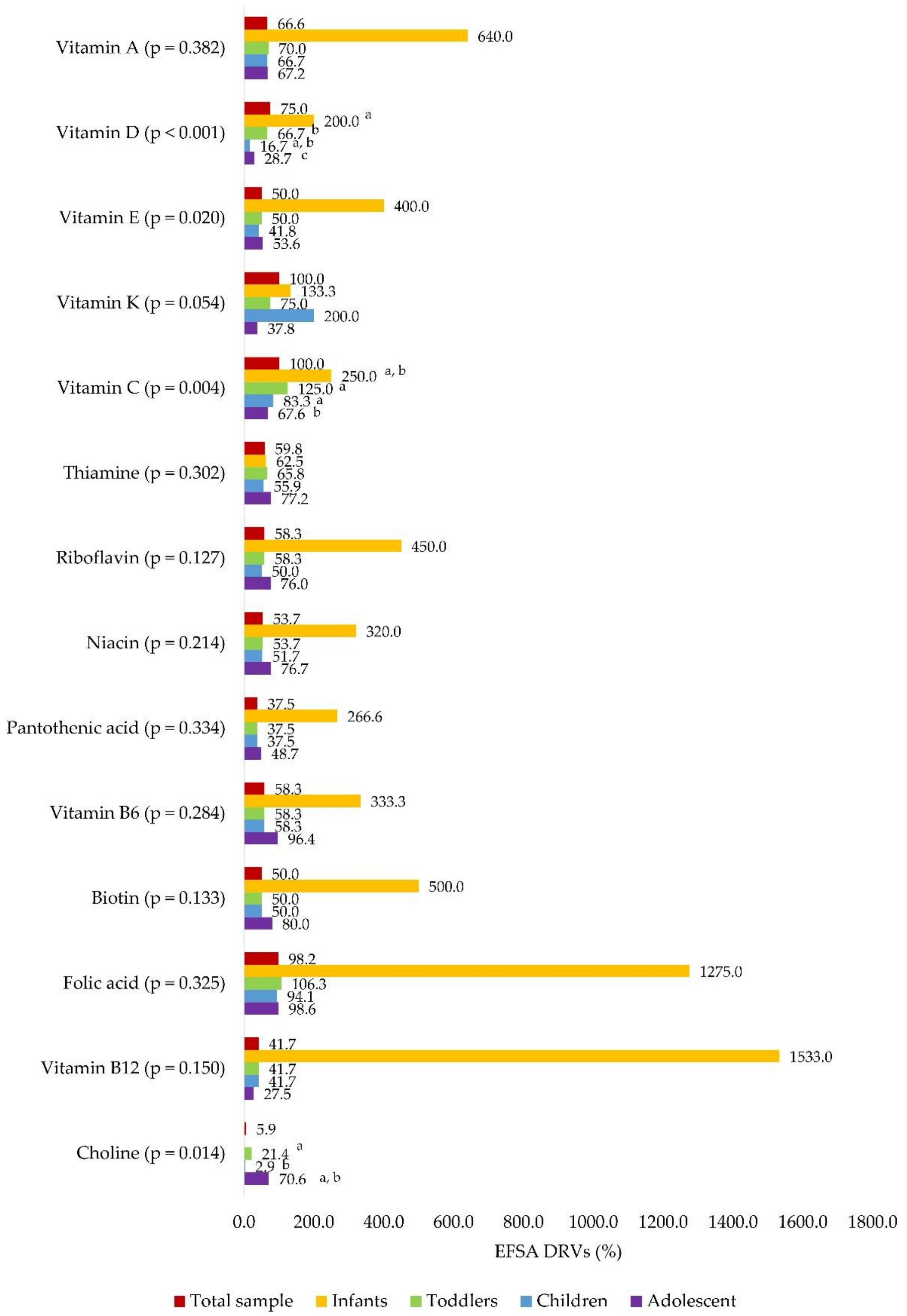

Figure S2. Contribution of vitamins from dietary supplements to EFSA DRVs among supplement users in the Croatian pediatric population participating in the NIPNAD 2017–2021 and NIPNOD 2018–2023 surveys. * Age groups were compared using the Kruskal-Wallis test with post hoc Dunn's test (adjusted significance), where a, b indicated significant differences ($p < 0.05$).

Table S4. Unweighted prevalence of mineral use and daily mineral intake from dietary supplements among supplement users in the Croatian pediatric population participating in the NIPNAD 2017–2021 and NIPNOD 2018–2023 surveys.

Active compound	Total sample (N = 2078)	Infants (n = 322)	Toddlers (n = 535)	Children (n = 963)	Adolescents (n = 258)	*p* Value *
Calcium (mg), % [1]; M (IQR) [2]	*4.3;*<br>60.0 (30.0 – 60.0)	*0.0;*<br>-	*5.4;*<br>60.0 (30.0 – 60.0)	*5.5;*<br>32.0 (30.0 – 60.0)	*3.1;*<br>80.0 (45.0 – 200.0)	*< 0.001*<br>0.059
Chloride (mg), %; M (IQR)	*0.4;*<br>150.0 (122.5 – 216.3)	*0.0;*<br>-	*1.1;*<br>146.5 (102.0 – 214.5)	*0.0;*<br>-	*0.8;*<br>225.0 (150.0 – 300.0)	*0.002*<br>0.286
Chromium (μg); %; M (IQR)	*1.2;*<br>10.5 (5.2 – 84.0)	*0.3;*<br>160.0 (N/A)	*1.3;*<br>8.9 (6.6 – 11.0)	*1.3;*<br>11.0 (5.0 – 160.0)	*1.2;*<br>12.0 (6.1 – 18.0)	*0.475*<br>0.422
Copper (μg), %; M (IQR)	*1.3;*<br>103.5 (25.0 – 260.0)	*0.0;*<br>-	*2.2;*<br>87.0 (75.0 – 180.0) [a]	*1.1;*<br>87.0 (6.6 – 270.0) [a]	*1.2;*<br>500.0 (380.0 – 510.0) [b]	*0.024*<br>0.049
Fluoride (mg), %; M (IQR)	*0.4;*<br>0.3 (0.2 – 0.4)	*0.6;*<br>0.3 (N/A)	*0.9;*<br>0.2 (0.2 – 76.2)	*0.0;*<br>-	*0.8;*<br>0.3 (0.2 – 0.4)	*0.007*<br>0.929
Iodine (μg), %; M (IQR)	*5.3;*<br>25.0 (18.8 – 37.5)	*0.3;*<br>240.0 (-)	*6.2;*<br>19.0 (18.8 – 37.5)	*7.3;*<br>27.5 (18.8 – 37.5)	*2.3;*<br>31.8 (18.8 – 52.0)	*< 0.001*<br>0.206
Iron (mg), %; M (IQR)	*6.4;*<br>1.8 (0.9 – 7.0)	*6.2;*<br>50.0 (25.0 – 50.0) [a]	*7.9;*<br>1.8 (0.9 – 7.0) [b]	*6.5;*<br>1.5 (0.9 – 1.8) [b]	*3.1;*<br>2.6 (1.3 – 4.7) [b]	*0.085*<br>< 0.001
Magnesium (mg), %; M (IQR)	*1.3;*<br>51.3 (25.5 – 125.0)	*0.0;*<br>-	*1.5;*<br>26.5 (13.1 – 39.0) [a]	*0.7;*<br>32.0 (18.8 – 72.9) [a, b]	*4.3;*<br>125.0 (69.3 – 168.8) [b]	*< 0.001*<br>0.004
Manganese (mg), %; M (IQR)	*1.6;*<br>0.6 (0.4 – 1.2)	*0.3;*<br>3.0 (N/A)	*2.1;*<br>0.6 (0.4 – 0.8)	*2.0;*<br>0.6 (0.3 – 1.5)	*1.2;*<br>1.0 (0.8 – 1.0)	*0.128*<br>0.362
Molydbenum (μg), %; M (IQR)	*0.8;*<br>32.0 (16.0 – 150.0)	*0.3;*<br>150.0 (N/A)	*1.1;*<br>9.3 (8.0 – 16.0)	*0.7;*<br>150.0 (75.0 – 150.0)	*1.2;*<br>25.0 (20.5 – 28.5)	*0.551*<br>0.362
Phosphorus (mg), %; M (IQR)	*0.7;*<br>93.8 (55.0 – 135.7)	*0.0;*<br>-	*1.9;*<br>93.8 (55.0 – 135.7) [a, b]	*0.2;*<br>29.9 (4.7 – 55.0) [a]	*0.8;*<br>225.0 (150.0 – 300.0) [b]	*0.001*<br>0.038

Table S4. *Continued*

Potassium (mg), %; M (IQR)	*0.7;* 112.0 (5.0 – 247.5)	*0.0;* -	*1.5;* 138.5 (104.5 – 236.5) [a, b]	*0.4;* 3.8 (2.6 – 7.0) [a]	*0.8;* 600.0 (400.0 – 800.0) [b]	*0.037* 0.022
Selenium (µg), %; M (IQR)	*1.5;* 17.5 (8.8 – 45.0)	*0.3;* 90.0 (N/A)	*1.9;* 10.4 (5.0 – 17.5)	*1.7;* 18.8 (8.8 – 70.0)	*1.9;* 24.0 (12.0 – 25.0)	*0.193* 0.199
Sodium (mg), %; M (IQR)	*0.7;* 67.5 (7.5 – 150.0)	*0.6;* 0.3 (N/A) [a]	*1.3;* 90.0 (40.3 – 142.5) [a, b]	*0.3;* 7.5 (4.5 – 77.8) [a, b]	*0.8;* 225.0 (150.0 – 300.0) [b]	*0.126* 0.040
Zinc (mg), %; M (IQR)	*7.1;* 2.5 (1.3 – 4.4)	*0.3;* 10.0 (N/A)	*9.0;* 2.6 (1.3 – 4.0)	*9.6;* 2.5 (1.3 – 4.0)	*2.7;* 5.2 (3.8 – 9.0)	*< 0.001* 0.068

[1] Percentage of vitamin users; [2] Median (interquartile range); - no intake; N/A – interquartile range was not calculated for subgroups with very small sample sizes (n ≤ 2); * Age groups were compared using a chi-squared test or Fisher exact test (italic p Values), and the Mann-Whitney U test or Kruskal-Wallis test with post hoc Dunn's test (adjusted significance), where a,b, c indicated significant differences ($p < 0.05$).

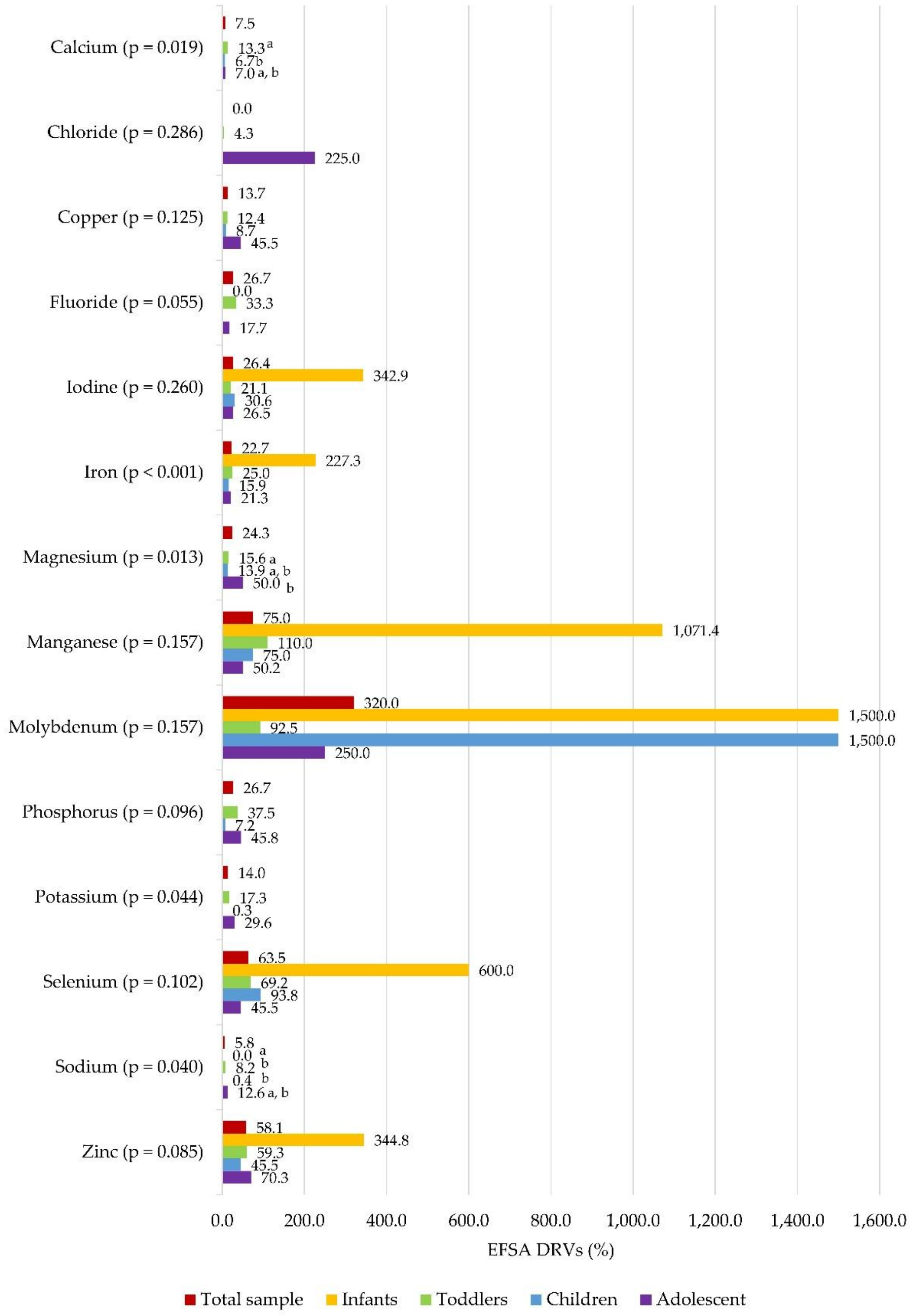

Figure S3. Contribution of minerals from dietary supplements to EFSA DRVs among supplement users in the Croatian pediatric population participating in the NIPNAD 2017–2021 and NIPNOD 2018–2023 surveys. * Age groups were compared using the Kruskal-Wallis test with post hoc Dunn's test (adjusted significance), where a, b indicated significant differences ($p < 0.05$).

Table S5. Unweighted prevalence of use and daily intake of active compounds from supplements containing proteins, amino acids, amino acid-related compounds, fatty acids, lipid formulations, prebiotics and functional carbohydrates among dietary supplement users in the Croatian pediatric population participating in the NIPNAD 2017–2021 and NIPNOD 2018–2023 surveys.

Active compound	Total sample (N = 2078)	Infants (n = 322)	Toddlers (n = 535)	Children (n = 963)	Adolescents (n = 258)	*p* Value *
Protein, amino acids and amino acid-related compounds						
Proteins (g), % [1]; M (IQR) [2]	*0.3;*<br>23.0 (12.3 – 23.5)	*0.0;*<br>-	*0.0;*<br>-	*0.0;*<br>-	*2.7;*<br>23.0 (12.3 – 23.5)	*< 0.001*<br>NC
Branched-chain amino acids (g), %; M (IQR)	*0.2;*<br>3.8 (2.5 – 5.0)	*0.0;*<br>-	*0.0;*<br>-	*0.0;*<br>-	*1.6;*<br>3.8 (2.5 – 5.0)	*< 0.001*<br>NC
Taurine (mg), %; M (IQR)	*0.2;*<br>9.0 (7.0 – 13.8)	*0.0;*<br>-	*0.7;*<br>9.0 (7.0 – 13.8)	*0.0;*<br>-	*0.0;*<br>-	*0.009*<br>NC
L-carnitine (mg), %; M (IQR)	*0.2;*<br>3.3 (2.0 – 23.8)	*0.0;*<br>-	*0.7;*<br>3.3 (2.0 – 23.8)	*0.0;*<br>-	*0.0;*<br>-	*0.009*<br>NC
L-carnosine (mg), %; M (IQR)	*0.1;*<br>125.0	*0.0;*<br>-	*0.2;*<br>125.0 (N/A)	*0.0;*<br>-	*0.0;*<br>-	*0.410*<br>NC
Fatty acids and lipid formulations						
Docosahexaenoic acid (mg); %; M (IQR)	*1.9;*<br>117.5 (25.2 – 450.0)	*0.6;*<br>15.0 (10.0 – 20.0)	*3.9;*<br>125.0 (25.3 – 600.0)	*1.6;*<br>300.0 (75.0 – 450.0)	*0.8;*<br>44.5 (34.0 – 55.0)	*0.001*<br>0.191
Eicosapentaenoic acid (mg), %; M (IQR)	*1.4;*<br>200.0 (25.0 – 400.0)	*0.0;*<br>-	*2.8;*<br>200.0 (21.9 – 400.0)	*1.3;*<br>200.0 (165.0 – 400.0)	*0.8;*<br>44.3 (6.0 – 82.5)	*0.006*<br>0.325
Docosahexaenoic + Eicosapentaenoic acid (mg), %	*0.1*	*0.0*	*0.4*	*0.0*	*0.0*	*0.123*
Arachidonic acid (mg), %; M (IQR)	*0.2;*<br>5.6 (3.7 – 25.6)	*0.0;*<br>-	*0.9;*<br>5.6 (3.7 – 25.6)	*0.0;*<br>-	*0.0;*<br>-	*0.002*<br>NC
Other omega-3 fatty acids (mg), %; M (IQR)	*1.0;*<br>225.0 (112.5 – 225.0)	*0.0;*<br>-	*1.7;*<br>225.0 (112.5 – 225.0)	*1.0;*<br>168.8 (112.5 – 225.0)	*0.4;*<br>27.5 (N/A)	*0.072*<br>0.175
Fish oil (mg), %; M (IQR)	*0.2;*<br>287.5 (56.3 – 750.0)	*0.0;*<br>-	*0.0;*<br>-	*0.3;*<br>500.0 (268.8 – 750.0)	*0.4;*<br>75.0 (N/A)	*0.411*<br>NC
Rapeseed oil (ml), %; M (IQR)	*0.1;*<br>0.5 (N/A)	*0.3;*<br>0.5 (N/A)	*0.2;*<br>0.5 (N/A)	*0.0;*<br>-	*0.0;*<br>-	*0.366*<br>NC

Table S5. *Continued*

Prebiotic and functional carbohydrate compounds						
Inulin, %	*0.1*	*0.0*	*0.0*	*0.3*	*0.0*	*0.342*
Fructooligosaccharides, %	*0.1*	*0.0*	*0.2*	*0.2*	*0.0*	*0.759*
Lactulose (g), %); M (IQR)	*0.05;*<br>10.0 (N/A)	*0.0;*<br>-	*0.2;*<br>10.0 (N/A)	*0.0;*<br>-	*0.0;*<br>-	*0.410*<br>NC
Inositol (mg), %; M (IQR)	*0.9;*<br>5.0 (0.01 – 10.0)	*0.0;*<br>-	*1.3;*<br>10.0 (7.5 – 17.5)	*1.1;*<br>0.5 (0.01 – 5.0)	*0.0;*<br>-	*0.068*<br>0.035
Beta-glucan (mg), %; M (IQR)	*4.3;*<br>25.0 (17.5 – 42.5)	*0.0;*<br>-	*6.2;*<br>20.0 (20.0 – 40.0)	*5.5;*<br>25.0 (15.0 – 50.0)	*1.2;*<br>40.0 (22.5 – 470.0)	*< 0.001*<br>0.771
Immunoglucan (mg), %; M (IQR)	*1.5;*<br>5.0 (5.0 – 10.0)	*0.0;*<br>-	*1.9;*<br>7.5 (5.0 – 10.0)	*2.2;*<br>5.0 (5.0 – 10.0)	*0.0;*<br>-	*0.006*<br>0.775
Arabinoxylan rice bran (mg), %; M (IQR)	*0.05;*<br>1000.0 (N/A)	*0.0;*<br>-	*0.2;*<br>1000.0 (N/A)	*0.0;*<br>-	*0.0;*<br>-	*0.410*<br>NC

[1] Percentage of vitamin users; [2] Median (interquartile range); "-"no intake; N/A – interquartile range was not calculated for subgroups with very small sample sizes ($n \leq 2$); NC – p Value was not calculated as low use of dietary supplements was identified; * Age groups were compared using a chi-squared test or Fisher exact test (italic p Values), and the Kruskal-Wallis test or Mann-Whitney U test ($p < 0.05$).

Table S6. Unweighted prevalence of probiotic species use among the Croatian pediatric population participating in the NIPNAD–2017–2021 and NIPNOD–2018–2023 surveys.

Dietary supplement	Total sample (N = 2078)	Infants (n = 322)	Toddlers (n = 535)	Children (n = 963)	Adolescents (n = 258)	*p* Value *
Lactobacillus delbrueckii subsp. bulgaricus [1] (%)	0.2	0.0	0.4	0.2	0.4	0.601
Lactobacillus acidophilus [2] (%)	0.8	0.0	1.5	0.7	0.4	0.093
Lactobacillus gasseri [3] (%)	< 0.1	0.0	0.2	0.0	0.0	0.537
Lactobacillus helveticus [4] (%)	0.2	0.3	0.2	0.2	0.4	0.788
Lacticaseibacillus casei [5] (%)	0.2	0.0	0.4	0.2	0.4	0.601
Lacticaseibacillus paracasei [6] (%)	0.1	0.0	0.0	0.2	0.0	0.761
Lacticaseibacillus rhamnosus [7] (%)	6.2	5.0	9.5	6.0	1.6	< 0.001
Ligilactobacillus salivarius [8] (%)	0.2	0.0	0.2	0.2	0.4	0.709
Lactiplantibacillus plantarum [9] (%)	0.5	0.0	0.6	0.6	0.4	0.655
Limosilactobacillus reuteri [10] (%)	2.4	9.0	3.2	0.4	0.0	< 0.001
Lactococcus lactis [11] (%)	0.9	0.9	1.3	0.8	0.4	0.683
Bifidobacterium lactis [12] (%)	4.6	0.9	6.4	5.7	1.2	< 0.001
Bifidobacterium bifidum [13] (%)	1.0	0.9	1.5	0.9	0.4	0.506
Bifidobacterium breve [14] (%)	0.5	0.3	0.9	0.3	0.4	0.394
Bifidobacterium longum [15] (%)	0.5	0.6	0.7	0.3	0.4	0.589
Bifidobacterium infantis [16] (%)	0.4	0.0	0.6	0.4	0.4	0.683
Streptococcus thermophilus [17] (%)	0.4	0.3	0.6	0.4	0.4	0.959
Bacillus subtilis [18] (%)	0.1	0.0	0.0	0.2	0.4	0.343
Bacillus coagulans [19] (%)	0.3	0.0	0.6	0.3	0.0	0.564
Saccharomyces cerevisiae [20] (%)	0.1	0.0	0.2	0.2	0.0	0.999
Saccharomyces boulardii [21] (%)	0.3	0.6	0.4	0.3	0.0	0.736

[1] Included strain PXN 39; [2] Included strains are LA-5, PXN 35, LA-14®, Lac361, and products with unspecified strains; [4] Included strains are LA 102 and PXN 45; [5] Included strain is PXN 37; [7] Included strains are GG (ATCC 53103), HN001™, PXN 54 and LA 801; [8] Included strain is PXN 57, and products with unspecified strains; [9] Included strains are PXN 47, LP115®, and products with unspecified strains; [10] Included strain is DSM 17938; [11] Included strains are W58, LA 10 and susp. lactis PXN 63; [12] Included strains W51, W52, BL04, HN019™, BI-04® and BB-12; [13] Included strains are W23, BB06 and PXN 23; [14] Included strains are BB03, PXN 25 and M-16V; [15] Included strains are BL05, LA 101, PXN 30, BB536, and products with unspecified strains; [16] Included strain PXN 27, and products with unspecified strains; [17] Included strains are LA 104, PXN 66, and products with unspecified strains; [18] Included strain is PXN 21; [19] Included strain is MTCC 5260 ®; [20] Reported as plasmolyzed yeast preparation; [21] Included strain I-3799. [3,6] Only products with unspecified strains are included; * Age groups were compared using a chi-squared test or Fisher exact test ($p < 0.05$).

Table S7. Unweighted prevalence of bee-produce formulations use among the Croatian pediatric population participating in the NIPNAD 2017–2021 and NIPNOD 2018–2023 surveys

Dietary supplement	Total sample (N = 2078)	Infants (n = 322)	Toddlers (n = 535)	Children (n = 963)	Adolescents (n = 258)	*p* Value*
Honey (%)	1.7	0.0	1.7	0.0	0.0	0.020
Bee pollen (%)	1.0	0.0	0.9	1.6	0.0	0.027
Royal jelly (%)	0.6	0.0	0.4	0.7	1.2	0.244
Propolis (%)	1.3	0.0	1.3	2.0	0.0	0.010
10-hydroxy-2-decenoic acid (%)	0.3	0.0	0.4	0.6	0.0	0.352

* Age groups were compared using a Fisher exact test ($p < 0.05$).

Table S8. Unweighted prevalence of digestive enzymes use among the Croatian pediatric population participating in the NIPNAD 2017–2021 and NIPNOD 2018–2023 surveys

Dietary supplement	Total sample (N = 2078)	Infants (n = 322)	Toddlers (n = 535)	Children (n = 963)	Adolescents (n = 258)	*p* Value*
Lipase (%)	0.05	0.0	0.0	0.1	0.0	0.763
Amylase (%)	0.05	0.0	0.0	0.1	0.0	0.763
Protease 3.0 (%)	0.05	0.0	0.0	0.1	0.0	0.763
Protease 4.5 (%)	0.05	0.0	0.0	0.1	0.0	0.763
Protease 6.0 (%)	0.05	0.0	0.0	0.1	0.0	0.763
Peptidase (%)	0.05	0.0	0.0	0.1	0.0	0.763
Phytase (%)	0.05	0.0	0.0	0.1	0.0	0.763
Cellulase (%)	0.05	0.0	0.0	0.1	0.0	0.763
Lactulose (%)	0.05	0.0	0.2	0.0	0.0	0.410
Bromelain (%)	0.1	0.0	0.1	0.0	0.4	0.384
Papain (%)	0.1	0.0	0.1	0.0	0.4	0.384

* Age groups were compared using a Fisher exact test ($p < 0.05$).

Table S9. Unweighted prevalence of phytochemicals formulation use among the Croatian pediatric population participating in the NIPNAD 2017–2021 and NIPNOD 2018–2023 surveys

Dietary supplement	Total sample (N = 2078)	Infants (n = 322)	Toddlers (n = 535)	Children (n = 963)	Adolescents (n = 258)	*p* Value*
Polyphenols (%)	0.05	0.0	0.0	0.1	0.0	0.763
Citrus bioflavonoids (%)	0.1	0.0	0.0	0.6	0.0	0.018
Galangin (%)	0.05	0.0	0.0	0.1	0.0	0.763
Cichoric acid (%)	0.05	0.0	0.1	0.0	0.0	0.793
Propolis flavonoids (%)	0.5	0.0	0.8	0.6	0.0	0.219
Acetogenin (%)	0.05	0.0	0.1	0.0	0.0	0.793
Curcumin (%)	0.05	0.0	0.1	0.0	0.0	0.793

* Age groups were compared using a Fisher exact test ($p < 0.05$).

Table S10. Unweighted prevalence of other active compound use among the Croatian pediatric population participating in the NIPNAD 2017–2021 and NIPNOD 2018–2023 surveys

Dietary supplement	Total sample (N = 2078)	Infants (n = 322)	Toddlers (n = 535)	Children (n = 963)	Adolescents (n = 258)	*p* Value*
Coenzyme Q10 (%)	0.2	0.0	0.1	0.2	0.8	0.101
Lecithin (%)	0.2	0.0	0.0	1.1	0.0	0.001
Citrate (%)	0.05	0.0	0.1	0.0	0.0	0.793
Colostrum (18% IGg) (%)	0.05	0.0	0.1	0.0	0.0	0.793
Spirulina (%)	0.1	0.0	0.0	0.1	0.1	0.375
Boswellia (%)	0.05	0.0	0.1	0.0	0.0	0.793

* Age groups were compared using a Fisher exact test ($p < 0.05$).

Table S11. Unweighted prevalence of essential oil use among the Croatian pediatric population participating in the NIPNAD 2017–2021 and NIPNOD 2018–2023 surveys

Dietary supplement	Total sample (N = 2078)	Infants (n = 322)	Toddlers (n = 535)	Children (n = 963)	Adolescents (n = 258)	*p* Value*
Eucalyptus (%)	0.05	0.0	0.1	0.0	0.0	0.793
Peppermint (%)	0.05	0.0	0.1	0.0	0.0	0.793
Black seeds (%)	0.1	0.0	0.1	0.0	0.4	0.384
Thyme (%)	0.05	0.0	0.1	0.0	0.0	0.793

* Age groups were compared using a Fisher exact test ($p < 0.05$).

Table S12. Unweighted prevalence of plant powders, concentrates and whole-food preparations use among the Croatian pediatric population participating in the NIPNAD 2017–2021 and NIPNOD 2018–2023 surveys

Dietary supplement	Total sample (N = 2078)	Infants (n = 322)	Toddlers (n = 535)	Children (n = 963)	Adolescents (n = 258)	*p* Value*
Cocoa powder (%)	0.2	0.0	0.5	0.0	0.0	0.159
Cinnamon powder (%)	0.2	0.0	0.5	0.0	0.0	0.159
Acai berry powder (%)	0.2	0.0	0.5	0.0	0.0	0.159
Wheatgrass powder (%)	0.2	0.0	0.5	0.0	0.0	0.159
Fruit and vegetable powder [1] (%)	0.1	0.0	0.0	0.4	0.0	0.081
Goji berry powder (%)	0.1	0.0	0.0	0.2	0.0	0.509
Alfalfa leaf powder (%)	0.05	0.0	0.0	0.1	0.0	0.763
Apple juice powder (%)	0.05	0.0	0.0	0.1	0.0	0.763
Grain concentrate blend [2] (%)	0.2	0.0	0.2	0.3	0.0	0.608

[1] carrot, beetroot, strawberry, apple, broccoli, apricot; [2] wheat germ, rice bran and sprout, and soy powder; * Age groups were compared using a Fisher exact test ($p < 0.05$).

Table S13. Unweighted prevalence of plant extracts use among the Croatian pediatric population participating in the NIPNAD 2017–2021 and NIPNOD 2018–2023 surveys

Dietary supplement	Total sample (N = 2078)	Infants (n = 322)	Toddlers (n = 535)	Children (n = 963)	Adolescents (n = 258)	p Value*
Peppermint leaf (%)	0.5	0.0	0.2	1.0	0.0	0.029
Echinacea (%)	0.2	0.0	0.4	0.3	0.0	0.573
Rosehip (%)	0.6	0.0	0.4	1.1	0.0	0.040
Elderberry (%)	1.7	0.0	1.7	2.7	0.0	< 0.001
Acerola (%)	0.3	0.0	0.2	0.5	0.0	0.302
Carrot (%)	0.2	0.0	0.0	0.5	0.0	0.122
Pineapple (%)	0.05	0.0	0.0	0.1	0.0	0.763
Fennel root (%)	0.05	0.0	0.0	0.1	0.0	0.763
Dandelion root (%)	0.1	0.0	0.0	0.2	0.0	0.501
Parsley root (%)	0.05	0.0	0.0	0.1	0.0	0.763
Ginger (%)	0.05	0.0	0.0	0.1	0.0	0.763
Lemon balm leaf (%)	0.05	0.0	0.0	0.1	0.0	0.763
Gentian root (%)	0.05	0.0	0.0	0.1	0.0	0.763
Grapefruit seed (%)	0.1	0.0	0.2	0.0	0.0	0.759
Cistus incanus leaf (%)	0.4	0.3	0.2	0.6	0.0	0.389
Olive leaf (%)	0.2	0.0	0.0	0.4	0.0	0.200
Avocado (%)	0.2	0.0	0.0	0.4	0.0	0.200
Mallow root (%)	0.1	0.0	0.2	0.2	0.0	0.759
Sage leaf (%)	0.1	0.0	0.2	0.2	0.0	0.759
Grape seed (%)	0.05	0.0	0.2	0.0	0.0	0.410
Anis (%)	0.4	0.0	0.2	0.8	0.0	0.078
Spruce shoot (%)	0.05	0.0	0.2	0.0	0.0	0.410
Astragalus root (%)	0.2	0.0	0.4	0.2	0.0	0.563
Everlasting flowers (%)	0.05	0.0	0.2	0.0	0.0	0.410
Fig and plum (%)	0.005	0.0	0.1	0.0	0.0	0.793
Juniper berry (%)	0.05	0.0	0.2	0.0	0.0	0.410
American cranberry (%)	0.0	0.0	0.0	0.1	0.0	0.763

* Age groups were compared using a Fisher exact test ($p < 0.05$).

Table S14. Crude binary logistic regression of sociodemographic, lifestyle and anthropometric characteristics as factors associated with use of dietary supplements in Croatian pediatric population participating in the NIPNAD 2017–2021 and NIPNOD 2018–2023 surveys.

Variables	**Infants (n = 322)**			**Toddlers (n = 535)**			**Children (n = 963)**			**Adolescents (n = 258)**		
	OR	95% CI	*p* Value *	OR	95% CI	*p* Value *	OR	95% CI	*p* Value *	OR	95% CI	*p* Value *
Sex:												
Boys							Ref.					
Girls	0.660	0.399 – 1.093	0.106	1.093	0.778 – 1.536	0.607	0.857	0.644 – 1.141	0.291	0.776	0.375 – 1.603	0.492
Region:												
Dalmatia	0.532	0.247 – 1.146	0.107	0.679	0.420 – 1.099	0.115	1.043	0.700 – 1.554	0.837	1.162	0.411 – 3.287	0.777
Istra, Primorje and Gorski Kotar	0.580	0.243 – 1.388	0.221	0.645	0.367 – 1.134	0.645	1.118	0.688 – 1.817	0.653	1.308	0.420 – 4.072	0.643
Lika and Banovina	0.086	0.028 – 0.265	< 0.001	0.435	0.170 – 1.113	0.435	0.240	0.100 – 0.577	0.001	0.000	-	0.998
Northern Croatia	1.007	0.369 – 2.753	0.989	0.896	0.484 – 1.1658	0.896	0.750	0.468 – 1.203	0.234	0.586	0.195 – 1.760	0.341
Slavonia	0.999	0.481 – 2.076	0.998	0.739	0.452 – 1.207	0.739	0.889	0.583 – 1.354	0.583	0.944	0.289 – 3.082	0.925
Zagreb							Ref.					
Parents education level [1]:												
Low (ISCED 0-2)							Ref.					
Medium (ISCED 3-4)	-	-	0.999	-	-	0.999	-	-	0.999	1.000	0.204 – 4.911	0.999
High (ISCED 5-8)	1.690	0.953 – 2.997	0.072	1.304	0.828 – 2.053	0.252	1.399	0.995 – 1.967	0.054	0.975	0.461 – 2.063	0.947
Parents employment status:												
Employed for pay or profit							Ref.					
Student	0.578	0.052 – 6.491	0.657	1.120	0.156 – 8.018	0.910	0.662	0.074 – 5.950	0.712	2.734	0.669 – 11.176	0.162
Unemployed	0.578	0.140 – 2.383	0.449	0.672	0.159 – 2.884	0.589	1.323	0.395 – 4.436	0.650	0.000	-	0.999
Maternity leave or sick	0.289	0.018 – 4.694	0.383	0.224	0.026 – 1.932	0.174	0.441	0.098 – 1.986	0.286	0.000	-	0.999
Housewife	0.434	0.224 – 0.842	0.014	0.814	0.467 – 1.421	0.470	0.000	-	0.998	0.000	-	0.999
Retired							0.834	0.509 – 1.365	0.470	0.580	0.129 – 2.598	0.476
Household income NIPNAD 2017-2021 [2]:												
< 378 €							Ref.					
378–757 €	0.000	-	0.999	-	-	0.999	0.000	-	0.999			
758–1141 €	1.339	0.371 – 4.840	0.656	3.316	0.912 – 12.053	0.069	2.542	0.703 – 9.199	0.155			
1142–1513 €	1.187	0.343 – 4.110	0.786	2.314	0.707 – 7.577	0.166	2.200	0.631 – 7.668	0.216			
>1 513 €	2.260	0.700 – 7.297	0.173	2.693	0.850 – 8.530	0.092	3.171	0.936 – 10.742	0.064			
Household income NIPNOD 2018-2023 [2]:											Ref.	
< 451 €										0.000	-	0.999
451 – 902 €										0.196	0.034 – 1.115	0.066
903 – 1354 €										0.598	0.155 – 2.311	0.456
1355 – 1805 €												
Presence of chronic disease:												
No							Ref.					
Yes	2.067	0.693 – 6.165	0.193	2.574	1.442 – 4.596	0.001	1.091	0.701 – 1.699	0.699	3.305	1.371 – 7.968	0.008
Physical activity level [3]:												
Low										Ref.		
Medium							1.154	0.458 – 2.905	0.761	1.000	0.356 – 2.812	0.998
High							1.716	0.678 – 4.345	0.255	2.174	0.986 – 4.886	0.060

Table S14. *Continued*

Type of diet:												
Normal diet							Ref.					
Other dietary patterns [4]	5.400	0.702 – 41.534	0.105	3.474	1.348 – 8.954	0.010	2.041	1.055 – 3.951	0.034	4.580	1.661 – 12.633	0.003
Infants feeding habits [5]:												
Breastfeeding		Ref.										
Formula use	1.021	0.577 – 1.806	0.944									
Mix diet	1.596	0.813 – 3.132	0.174									
Energy intake	1.001	1.000 – 1.002	0.147	0.999	0.998 – 1.000	0.004	1.000	0.999 – 1.000	0.540	1.001	1.000 – 1.001	0.072
z-score of body mass index for age	0.811	0.659 – 0.997	0.046	0.872	0.761 – 0.998	0.047	0.957	0.862 – 1.062	0.407	0.739	0.534 – 1.024	0.069
Birth weight [5]:												
< 2500 g	2.913	0.358 – 23.704	0.318									
2500 – 4000 g		Ref.										
> 4000 g	1.254	0.569 – 2.764	0.574									

[1] ISCED - the International Standard Classification of Education; [2] During study official currency in Croatia was Croatian Kuna (1 EUR = 7.53450 HRK); [3] Surveyed in children and adolescents; [4] Includes vegetarian, slimming, gluten-free, diabetic, and elimination diets for food allergies, as well as unspecified diets and diets for unspecified health conditions. [5] Surveyed in infants; - cannot be calculated; blank cells – no data; * Odds ratios with corresponding 95% confidence intervals from binary logistic regression models.
